# Association Between Changes in Alcohol Consumption and Cancer Risk

**DOI:** 10.1001/jamanetworkopen.2022.28544

**Published:** 2022-08-24

**Authors:** Jung Eun Yoo, Kyungdo Han, Dong Wook Shin, Dahye Kim, Bong-seong Kim, Sohyun Chun, Keun Hye Jeon, Wonyoung Jung, Jinsung Park, Jin Ho Park, Kui Son Choi, Joo Sung Kim

**Affiliations:** 1 Department of Family Medicine, Healthcare System Gangnam Center, Seoul National University Hospital, Seoul, Republic of Korea; 2 Department of Statistics and Actuarial Science, Soongsil University, Seoul, Republic of Korea; 3 Department of Supportive Care Center/Department of Family Medicine, Samsung Medical Center, Seoul, Republic of Korea; 4 Department of Clinical Research Design and Evaluation, Samsung Advanced Institute for Health Science & Technology (SAIHST), Sungkyunkwan University, Seoul, Republic of Korea; 5 Department of Digital Health, SAIHST, Sungkyunkwan University, Seoul, Republic of Korea; 6 Department of Medical Statistics, The Catholic University of Korea, Seoul, Republic of Korea; 7 International Healthcare Center, Samsung Medical Center, Seoul, Republic of Korea; 8 Department of Family Medicine, Cha Gumi Medical Center, Cha University, Gumi-si, Gyeongsangbuk-do, Republic of Korea; 9 Department of Urology, Uijeongbu Eulji Medical Center, Eulji University, Uijeongbu-si, Gyeonggi-do, Republic of Korea; 10 Department of Family Medicine, Seoul National University Hospital, Seoul National University College of Medicine, Seoul, Republic of Korea; 11 Department of Cancer Control and Population Health, Graduate School of Cancer Science and Policy, National Cancer Center, Goyang, Republic of Korea; 12 Department of Internal Medicine and Liver Research Institute, Seoul National University College of Medicine, Seoul, Republic of Korea

## Abstract

**Importance:**

Although numerous studies have shown an association between alcohol consumption and cancer, how changes in drinking behavior increase or decrease the incidence of cancer is not well understood.

**Objective:**

To investigate the association between the reduction, cessation, or increase of alcohol consumption and the development of alcohol-related cancers and all cancers.

**Design, Setting, and Participants:**

This population-based cohort study analyzed adult beneficiaries in the Korean National Health Insurance Service. Participants (aged ≥40 years) included those who underwent a national health screening in both 2009 and 2011 and had available data on their drinking status. Data were analyzed from April 16 to July 6, 2020.

**Exposures:**

Alcohol consumption level, which was self-reported by participants in health screening questionnaires, was categorized into none (0 g/d), mild (<15 g/d), moderate (15-29.9 g/d), and heavy (≥30 g/d) drinking. Based on changes in alcohol consumption level from 2009 to 2011, participants were categorized into the following groups: nondrinker, sustainer, increaser, quitter, and reducer.

**Main Outcomes and Measures:**

The primary outcome was newly diagnosed alcohol-related cancers (including cancers of the head and neck, esophagus, colorectum, liver, larynx, and female breast), and the secondary outcome was all newly diagnosed cancers (except for thyroid cancer).

**Results:**

Among the 4 513 746 participants (mean [SD] age, 53.6 [9.6] years; 2 324 172 [51.5%] men), the incidence rate of cancer was 7.7 per 1000 person-years during a median (IQR) follow-up of 6.4 (6.1-6.6) years. Compared with the sustainer groups at each drinking level, the increaser groups had a higher risk of alcohol-related cancers and all cancers. The increased alcohol-related cancer incidence was associated with dose; those who changed from nondrinking to mild (adjusted hazard ratio [aHR], 1.03; 95% CI, 1.00-1.06), moderate (aHR, 1.10; 95% CI, 1.02-1.18), or heavy (aHR, 1.34; 95% CI, 1.23-1.45) drinking levels had an associated higher risk than those who did not drink. Those with mild drinking levels who quit drinking had a lower risk of alcohol-related cancer (aHR, 0.96; 95% CI, 0.92-0.99) than those who sustained their drinking levels. Those with moderate (aHR, 1.07; 95% CI, 1.03-1.12) or heavy (aHR, 1.07; 95% CI, 1.02-1.12) drinking levels who quit drinking had a higher all cancer incidence than those who sustained their levels, but when quitting was sustained, this increase in risk disappeared. Compared with sustained heavy drinking, reduced heavy drinking levels to moderate levels (alcohol-related cancer: aHR, 0.91 [95% CI, 0.86-0.97]; all cancers: aHR, 0.96 [95% CI, 0.92-0.99]) or mild levels (alcohol-related cancer: aHR, 0.92 [95% CI, 0.86-0.98]; all cancers: aHR, 0.92 [95% CI, 0.89-0.96]) were associated with decreased cancer risk.

**Conclusions and Relevance:**

Results of this study showed that increased alcohol consumption was associated with higher risks for alcohol-related and all cancers, whereas sustained quitting and reduced drinking were associated with lower risks of alcohol-related and all cancers. Alcohol cessation and reduction should be reinforced for the prevention of cancer.

## Key Points

**Question:**

How does the risk of developing cancer change after alcohol consumption is increased, stopped, or reduced?

**Findings:**

In this cohort study of 4 513 746 insured adults in Korea, those who increased their alcohol consumption had a higher risk for alcohol-related cancers and all cancers compared with those who had sustained levels of drinking, whereas those who reduced their alcohol consumption had a lower risk. Although an increased risk was observed temporarily after quitting drinking, no increased risk was observed when quitting was sustained.

**Meaning:**

Findings of this study suggest that drinking cessation and reduction should be reinforced for the prevention of cancer.

## Teaser

This cohort study examines the risk and incidence of alcohol-related cancers and all cancers among adults in Korea who self-reported their drinking behavior.

## Introduction

Cancer is the second leading cause of death globally, accounting for an estimated 9.6 million deaths in 2018.^1^ Alcohol consumption is the third major, modifiable cancer risk factor after tobacco use and excess body weight,^2^ and it is an established cause of at least 7 types of cancer.^3^

Although numerous studies have found an association between alcohol consumption and cancer,^4^ there is paucity of research into how the incidence of cancer increases or decreases with changes in drinking habits. Some studies of the association between alcohol cessation and risk of several cancers, including laryngeal or pharyngeal,^5^ esophageal,^6^ and liver cancers,^7^ reported reduced incidence. We found only 1 cohort study that reported an association between reduction in alcohol consumption and risk of cancer. The study found a modest decrease in the risk of upper digestive tract (oral cavity, pharynx, larynx, and esophagus) cancers among individuals who reduced their alcohol intake and an elevated risk among those who increased their alcohol intake.^8^

We conducted a cohort study to investigate the association between the reduction, cessation, or increase of alcohol consumption and the development of alcohol-related cancers and all cancers. Specifically, we measured alcohol consumption in a large cohort of Korean adults at 2 time points and incidence of cancer.

## Methods

This retrospective, population-based cohort study was approved by the Institutional Review Board of the Samsung Medical Center, which waived the informed consent requirement because the data were public and anonymized under confidentiality guidelines. This study was designed and conducted according to the Strengthening the Reporting of Observational Studies in Epidemiology (STROBE) reporting guideline.^9^

### Study Setting and Population

Korea has a mandatory social insurance system with insurance premiums that are determined by income level and not by health status. The National Health Insurance Service (NHIS) is a single insurer in Korea that covers approximately 97% of the population except for 3% of beneficiaries of the Medical Aid Program. Data on the use of medical facilities and records of prescriptions with *International Statistical Classification of Diseases and Related Health Problems, Tenth Revision* (*ICD-10*) diagnosis codes are gathered by the NHIS. In addition, the NHIS provides free biennial health screening for all beneficiaries older than 40 years and all employees regardless of age. This screening includes a self-administered questionnaire on health behavior (eg, medical history, smoking status, and drinking status), anthropometric measurements (eg, body mass index [calculated as weight in kilograms divided by height in meters squared] and blood pressure), and laboratory tests (eg, fasting glucose and lipid levels).^10^ The NHIS also collects information on demographic factors (eg, age, sex, place of residence, and income level) and links the data to a death registry database to manage the qualification of the enrollees. The NHIS database has been used to establish the cohort data for various epidemiologic studies.^11^

Using the NHIS database, we initially included in the present cohort 4 961 441 individuals (aged ≥40 years) who had available data on their drinking status in 2 consecutive biennial health screenings (2009 and 2011). Individuals were excluded if (1) they had a history of any cancer (n = 141 566) or cardiovascular disease (n = 65 108) before the 2011 health screening date; (2) they had any cancer (n = 40 982), any cardiovascular disease (n = 14 081), or died (n = 7881) within 1 year after the 2011 health screening date; or (3) they had any missing information (n = 178 077). Ultimately, 4 513 746 individuals were included in the primary analysis. These participants were followed up from 1 year after the 2011 health screening date to the date of incident cancer, death, or end of study (December 31, 2018), whichever occurred first (eFigure 1 in the Supplement).

### Exposure

Information on alcohol consumption was collected from the self-administered questionnaires at 2 separate health screenings in 2009 and 2011. The amount of pure alcohol intake per day was calculated from the drinking frequency per week and the typical amount consumed on each occasion. First, we grouped participants according to drinking levels based on their self-reported daily alcohol consumption: none (0 g/d), mild (<15 g/d), moderate (15-29.9 g/d), or heavy (≥30 g/d) drinking.^12,13^ Second, for the description of baseline characteristics and interpretation, we classified these participants into 5 groups based on their drinking levels between 2009 and 2011: (1) nondrinker, defined as sustained alcohol abstinence; (2) sustainer, defined as maintained baseline level of alcohol consumption; (3) increaser, defined as elevated level of alcohol consumption; (4) quitter, defined as stopping alcohol consumption from a baseline mild, moderate, or heavy level; or (5) reducer, defined as decreased level of alcohol consumption but not quitting.

### Outcomes

The primary outcome was newly diagnosed alcohol-related cancers, and the secondary outcome was all newly diagnosed cancers (*ICD-10* codes C00-C99) except for thyroid cancer (*ICD-10* code C73). Alcohol-related cancers were defined as established cancers, including cancers of the head and neck (oral cavity and pharynx; *ICD-10* codes C01-C10 and C12-14), esophagus (*ICD-10* code C15), colorectum (*ICD-10* codes C18-C20 but excluding appendix [*ICD-10* code C18.1]), liver (*ICD-10* code C22), larynx (*ICD-10* code C32), and female breast (*ICD-10* code C50) according to the list of cancers of the National Cancer Institute,^14^ World Cancer Research Fund and American Institute for Cancer Research,^4^ and International Agency for Research on Cancer.^15^ We excluded thyroid cancer from the definition of all cancers because it is a representative example of overdiagnosis by inadvertent thyroid cancer screening in Korea.^16,17^

To define cancer incidence, we used a special registration code in addition to the *ICD-10* diagnosis code. The NHIS has established a special co-payment reduction program to enhance health coverage and relieve the financial burden of patients with cancer. For example, patients pay only 5% of the total medical bill incurred for cancer-related medical care. Because enrollment in this co-payment reduction program is indicated by a special co-payment reduction code for cancer (V193) and requires a medical certificate from a physician, the cancer diagnoses included in this study are considered to be sufficiently reliable, and this method has been used in previous studies.^18,19^

We considered socioeconomic position, including income level and place of residence (urban or rural), to be a potential covariate. Household income was categorized into quartiles according to insurance premium levels, and those covered by the Medical Aid Program (the poorest 3% of the Korean population) were merged into the lowest income quartile. Smoking status was classified into never, former (<20 pack-years or ≥20 pack-years), or current smoker (<20 pack-years or ≥20 pack-years). Participants were also categorized according to whether they engaged in regular exercise. Regular exercise was defined as more than 30 minutes of moderate physical activity at least 5 times per week or more than 20 minutes of strenuous physical activity at least 3 times per week. Comorbidities (hypertension, diabetes, chronic kidney disease, and chronic obstructive pulmonary disease) were based on claims data before the screening date and health screening test results. To assess the overall comorbidity load, we used the primary care equivalent of the Charlson Comorbidity Index.

### Statistical Analysis

The association between changes in the drinking level and the incidence of cancer was estimated using a Cox proportional hazards regression model initially (crude model [model 1]) and then using a multivariable-adjusted model (model 2; adjusted for age, sex, socioeconomic position, smoking status, physical activity, comorbidities, and Charlson Comorbidity Index). To observe the associations between changes in drinking level and cancer incidence, we selected the sustainer group (no change from baseline) for each alcohol consumption level as the reference group. Stratified analysis was performed by age, sex, and smoking status in 2009.

For the secondary analysis, we explored the association of changes in the drinking level that occurred during the follow-up. We selected 3 542 927 participants (78.5%) whose health screening data were available for 2013. Using information on drinking levels at 3 consecutive health screenings, we repeated the same analysis of drinking level change from 2009 to 2011 to 2013.

All statistical analyses were performed using the SAS statistical package, version 9.4 (SAS Institute Inc). A 2-sided *P* < .05 was considered to be statistically significant. Data were analyzed from April 16 to July 6, 2020.

## Results

The 4 513 746 participants in the cohort had a mean (SD) age of 53.6 (9.6) years and included 2 324 172 men (51.5%) and 2 189 574 women (48.5%). From 2009 to 2011, 26.6% of participants with mild drinking, 9.6% with moderate drinking, and 8.6% with heavy drinking levels quit drinking. Compared with the quitter group, the increaser group tended to be younger, be male, have higher incomes, be current smokers, not engaged in regular exercise, and have a lower Charlson Comorbidity Index (Table 1).

**Table 1.** Characteristics of Study Participants According to Changes in Drinking Level Between 2009 and 2011

Characteristic	Participant drinking level, No. (%)				
	Nondrinker group (n = 2 218 002)	Quitter group (n = 377 325)	Reducer group (n = 302 732)	Sustainer group (n = 990 873)	Increaser group (n = 624 814)
Age, mean (SD), y	55.9 (10.3)	53.2 (9.9)	51.2 (8.8)	50.6 (8.7)	51.4 (9.1)
Sex					
Female	1 622 546 (73.2)	161 250 (42.7)	26 903 (8.9)	202 492 (20.4)	176 383 (28.2)
Male	595 456 (26.9)	216 075 (57.3)	275 829 (91.1)	788 381 (79.6)	448 431 (71.8)
Income level by quartile					
Quartile 1 (lowest)	529 519 (23.9)	84 178 (22.3)	52 584 (17.4)	178 708 (18.0)	125 231 (20.0)
Quartile 2	429 539 (19.4)	72 359 (19.2)	53 326 (17.6)	168 921 (17.1)	113 589 (18.2)
Quartile 3	528 717 (23.8)	90 324 (23.9)	79 150 (26.2)	242 315 (24.5)	152 820 (24.5)
Quartile 4 (highest)	730 227 (32.9)	130 464 (34.6)	117 672 (38.9)	400 929 (40.5)	233 174 (37.3)
Urban place of residence	978 883 (44.1)	171 834 (45.5)	139 161 (46.0)	474 503 (47.9)	284 642 (45.6)
Smoking status					
Never	1 858 340 (83.8)	218 936 (58.0)	75 193 (24.8)	370 041 (37.3)	326 109 (52.2)
Former					
<20 Pack-years	105 598 (4.8)	46 639 (12.4)	53 513 (17.7)	181 246 (18.3)	80 489 (12.9)
≥20 Pack-years	70 497 (3.2)	26 806 (7.1)	36 332 (12.0)	93 257 (9.4)	46 251 (7.4)
Current					
<20 Pack-years	79 152 (3.6)	40 049 (10.6)	56 010 (18.5)	162 259 (16.4)	82 311 (13.2)
≥20 Pack-years	104 415 (4.7)	44 895 (11.9)	81 684 (27.0)	184 070 (18.6)	89 654 (14.4)
Regular exercise	442 596 (20.0)	88 107 (23.4)	76 842 (25.4)	241 280 (24.4)	138 972 (22.2)
BMI, mean (SD)	23.9 (3.1)	24.0 (4.9)	24.3 (6.0)	24.0 (2.8)	24.1 (2.9)
Comorbidities					
Hypertension	236 908 (10.7)	40 014 (10.6)	36 792 (12.2)	96 711 (9.8)	64 681 (10.4)
Diabetes	770 554 (34.8)	123 141 (32.6)	110 233 (36.4)	309 989 (31.3)	199 546 (32.0)
Dyslipidemia	552 048 (24.9)	78 497 (20.8)	60 629 (20.0)	186 462 (18.8)	122 018 (19.5)
CKD	290 413 (13.1)	42 905 (11.4)	27 260 (9.0)	93 833 (9.5)	59 704 (9.6)
COPD	108 370 (4.9)	15 511 (4.1)	9629 (3.2)	30 654 (3.1)	22 245 (3.6)
CCI, mean (SD)	1.2 (1.4)	1.1 (1.3)	0.9 (1.2)	0.8 (1.2)	0.9 (1.3)

Abbreviations: BMI, body mass index (calculated as weight in kilograms divided by height in meters squared); CCI, Charlson Comorbidity Index; CKD, chronic kidney disease; COPD, chronic obstructive pulmonary disease.

### Alcohol Consumption Change and Cancer

We followed participants for a median (IQR) of 6.4 (6.1-6.6) years, yielding a total of 28 090 140 person-years. During this period, there were 215 676 cancer events (7.7 per 1000 person-years), 37.2% (80 263 cases) of which were alcohol-related cancers. Table 2 and eTable 1 in the Supplement show the associations between the change in alcohol consumption and risk of cancer, with the sustainer group at each drinking level as the reference group.

**Table 2.** Associations Between Changes in Drinking Level and Cancer

	Drinking level		No. (%)		Person-years	Cancer incidence rate per 1000 person-years	HR (95% CI)	
	2009	2011	Participants	Events			Model 1^a^	Model 2^b^
Alcohol-related cancers	None	None	2 218 002 (49.1)	39 765 (49.5)	13 985 909.0	2.8	1 [Reference]	1 [Reference]
	None	Mild	297 258 (6.6)	4958 (6.2)	1 873 661.5	2.6	0.93 (0.90-0.96)	1.03 (1.00-1.06)
	None	Moderate	43 165 (1.0)	816 (1.0)	270 934.5	3.0	1.06 (0.99-1.14)	1.10 (1.02-1.18)
	None	Heavy	24 761 (0.5)	577 (0.7)	154 205.1	3.7	1.32 (1.21-1.43)	1.34 (1.23-1.45)
	Mild	None	305 582 (6.8)	5425 (6.8)	1 923 062.0	2.8	1.15 (1.12-1.19)	0.96 (0.93-0.99)
	Mild	Mild	663 193 (14.7)	10 214 (12.7)	4 178 738.4	2.4	1 [Reference]	1 [Reference]
	Mild	Moderate	138 073 (3.1)	2358 (2.9)	867 957.0	2.7	1.11 (1.06-1.16)	1.10 (1.05-1.15)
	Mild	Heavy	41 864 (0.9)	856 (1.1)	261 888.9	3.3	1.34 (1.25-1.43)	1.17 (1.09-1.25)
	Moderate	None	44 370 (1.0)	984 (1.2)	276 633.2	3.6	1.35 (1.26-1.45)	1.05 (0.98-1.14)
	Moderate	Mild	160 850 (3.6)	2822 (3.5)	1 009 333.3	2.8	1.06 (1.01-1.12)	0.98 (0.93-1.04)
	Moderate	Moderate	177 446 (3.9)	2938 (3.7)	1 114 432.3	2.6	1 [Reference]	1 [Reference]
	Moderate	Heavy	79 693 (1.8)	1495 (1.9)	499 352.0	3.0	1.14 (1.07-1.21)	1.04 (0.98-1.11)
	Heavy	None	27 373 (0.6)	778 (1.0)	168 497.5	4.6	1.28 (1.18-1.38)	1.04 (0.96-1.12)
	Heavy	Mild	53 347 (1.2)	1149 (1.4)	332 919.1	3.5	0.95 (0.89-1.02)	0.92 (0.86-0.98)
	Heavy	Moderate	88 535 (2.0)	1736 (2.2)	553 395.5	3.1	0.87 (0.82-0.92)	0.91 (0.86-0.97)
	Heavy	Heavy	150 234 (3.3)	3392 (4.2)	937 483.6	3.6	1 [Reference]	1 [Reference]
All cancers (except thyroid)	None	None	2 218 002 (49.1)	104 645 (48.5)	13 834 229.0	7.6	1 [Reference]	1 [Reference]
	None	Mild	297 258 (6.6)	12 916 (6.0)	1 854 643.0	7.0	0.92 (0.90-0.94)	0.98 (0.97-1.00)
	None	Moderate	43 165 (1.0)	2267 (1.1)	267 413.2	8.5	1.12 (1.08-1.17)	0.98 (0.94-1.03)
	None	Heavy	24 761 (0.5)	1600 (0.7)	151 910.8	10.5	1.40 (1.33-1.47)	1.12 (1.07-1.18)
	Mild	None	305 582 (6.8)	14 624 (6.8)	1 901 404.6	7.7	1.11 (1.09-1.14)	0.98 (0.96-1.00)
	Mild	Mild	663 193 (14.7)	28 515 (13.2)	4 134 254.3	6.9	1 [Reference]	1 [Reference]
	Mild	Moderate	138 073 (3.1)	6512 (3.0)	858 167.6	7.6	1.10 (1.07-1.13)	1.02 (0.99-1.05)
	Mild	Heavy	41 864 (0.9)	2383 (1.1)	258 324.6	9.2	1.34 (1.28-1.40)	1.09 (1.04-1.13)
	Moderate	None	44 370 (1.0)	2852 (1.3)	272 309.1	10.5	1.41 (1.35-1.47)	1.07 (1.03-1.12)
	Moderate	Mild	160 850 (3.6)	7930 (3.7)	997 209.7	8.0	1.07 (1.04-1.10)	0.98 (0.95-1.02)
	Moderate	Moderate	177 446 (3.9)	8213 (3.8)	1 102 016.0	7.5	1 [Reference]	1 [Reference]
	Moderate	Heavy	79 693 (1.8)	4097 (1.9)	493 498.5	8.3	1.11 (1.07-1.16)	1.01 (0.97-1.05)
	Heavy	None	27 373 (0.6)	2143 (1.0)	165 607.5	12.9	1.32 (1.26-1.38)	1.07 (1.02-1.12)
	Heavy	Mild	53 347 (1.2)	3111 (1.4)	328 378.0	9.5	0.97 (0.93-1.01)	0.92 (0.89-0.96)
	Heavy	Moderate	88 535 (2.0)	4789 (2.2)	546 300.8	8.8	0.89 (0.86-0.93)	0.96 (0.92-0.99)
	Heavy	Heavy	150 234 (3.3)	9079 (4*.2*)	924 473.2	9.8	1 [Reference]	1 [Reference]

Abbreviation: HR, hazard ratio.

^a^ Model 1: crude model.

^b^ Model 2: adjusted for age, sex, socioeconomic position (income level and place of residence), smoking status, physical activity, comorbidities (hypertension, diabetes, dyslipidemia, chronic kidney disease, and chronic obstructive pulmonary disease), and Charlson Comorbidity Index.

**Figure 1.**
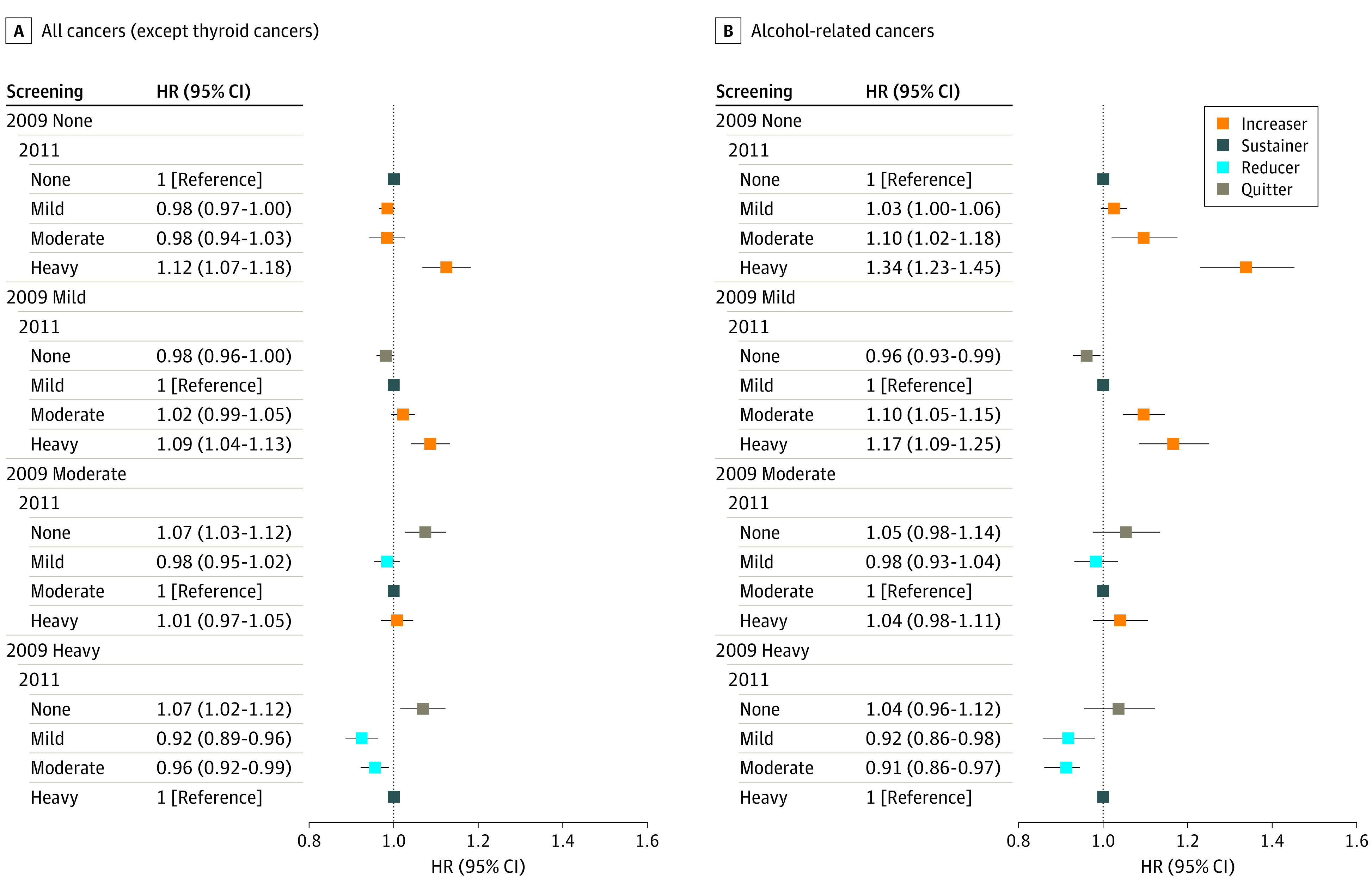
Risk of All Cancers and Alcohol-Related Cancers by Changes in Drinking Level Between 2009 and 2011 Hazard ratios (HRs) were adjusted for age, sex, socioeconomic position (income level and place of residence), smoking status, physical activity, comorbidities (hypertension, diabetes, dyslipidemia, chronic kidney disease, chronic obstructive pulmonary disease), and Charlson Comorbidity Index. Error bars indicate the 95% CIs.

Compared with the sustainer group, the increaser group had a higher risk of alcohol-related cancers, showing a dose-response association: nondrinking status was associated with increased risk of alcohol-related cancers as the status changed to mild drinking (adjusted hazard ratio [aHR], 1.03; 95% CI, 1.00-1.06), moderate drinking (aHR, 1.10; 95% CI, 1.02-1.18), or heavy drinking (aHR, 1.34; 95% CI, 1.23-1.45) (Table 2; Figure 1). Similarly, higher rates of alcohol-related cancers were found among those with a mild drinking level who changed to moderate (aHR, 1.10; 95% CI, 1.05-1.15) or heavy (aHR, 1.17; 95% CI, 1.09-1.25) drinking levels compared with those who sustained mild drinking levels. This pattern was also present for all cancers: increased risk was associated not only with nondrinking that became heavy drinking (aHR, 1.12; 95% CI, 1.07-1.18) but also mild drinking that became heavy drinking (aHR, 1.09; 95% CI, 1.04-1.13). When we examined specific cancer sites, we found that the increaser (from nondrinking) group had a high incidence of stomach, liver, gallbladder, and lung cancer; multiple myeloma; and leukemia (eTable 2 and eFigure 2 in the Supplement).

For alcohol-related cancers, moderate or heavy drinking that changed to nondrinking was not significantly associated with the increased rate (Table 2; Figure 1). Although not to a great magnitude, an association was found between quitting from moderate drinking (aHR, 1.07; 95% CI, 1.03-1.12) or heavy drinking levels (aHR, 1.07; 95% CI, 1.02-1.12) and increased risk of all cancers. The quitter group was associated with higher incidences of head and neck, esophagus, stomach, colorectum, liver, gallbladder, larynx, cervix uteri, and pancreas cancers compared with sustained nondrinking (eTable 2 and eFigure 2 in the Supplement). Such associations were most pronounced for esophageal cancers, with an aHR of 3.66 (95% CI, 2.77-4.83) for those with a heavy drinking level who stopped drinking.

The risk reduction for alcohol-related cancer was greater for those with a heavy drinking level than for those with a moderate or mild drinking level who decreased their alcohol consumption. Reduced drinking among participants with formerly heavy drinking levels was associated with lower rates of alcohol-related cancers, regardless of whether it became moderate drinking (aHR, 0.91; 95% CI, 0.86-0.97) or mild drinking (aHR, 0.92; 95% CI, 0.86-0.98) (Figure 1; Table 2). This association was consistent for all cancers. Heavy drinking that changed to moderate drinking (aHR, 0.96; 95% CI, 0.92-0.99) or mild (aHR, 0.92; 95% CI, 0.89-0.96) was associated with lower risks of alcohol-related cancers compared with sustained heavy drinking. For cancer site, participants who changed from heavy to moderate drinking levels had a lower incidence of breast, kidney, and gallbladder cancer than those who sustained a heavy drinking level (eTable 2 and eFigure 2 in the Supplement).

### Secondary Analysis

**Figure 2.**
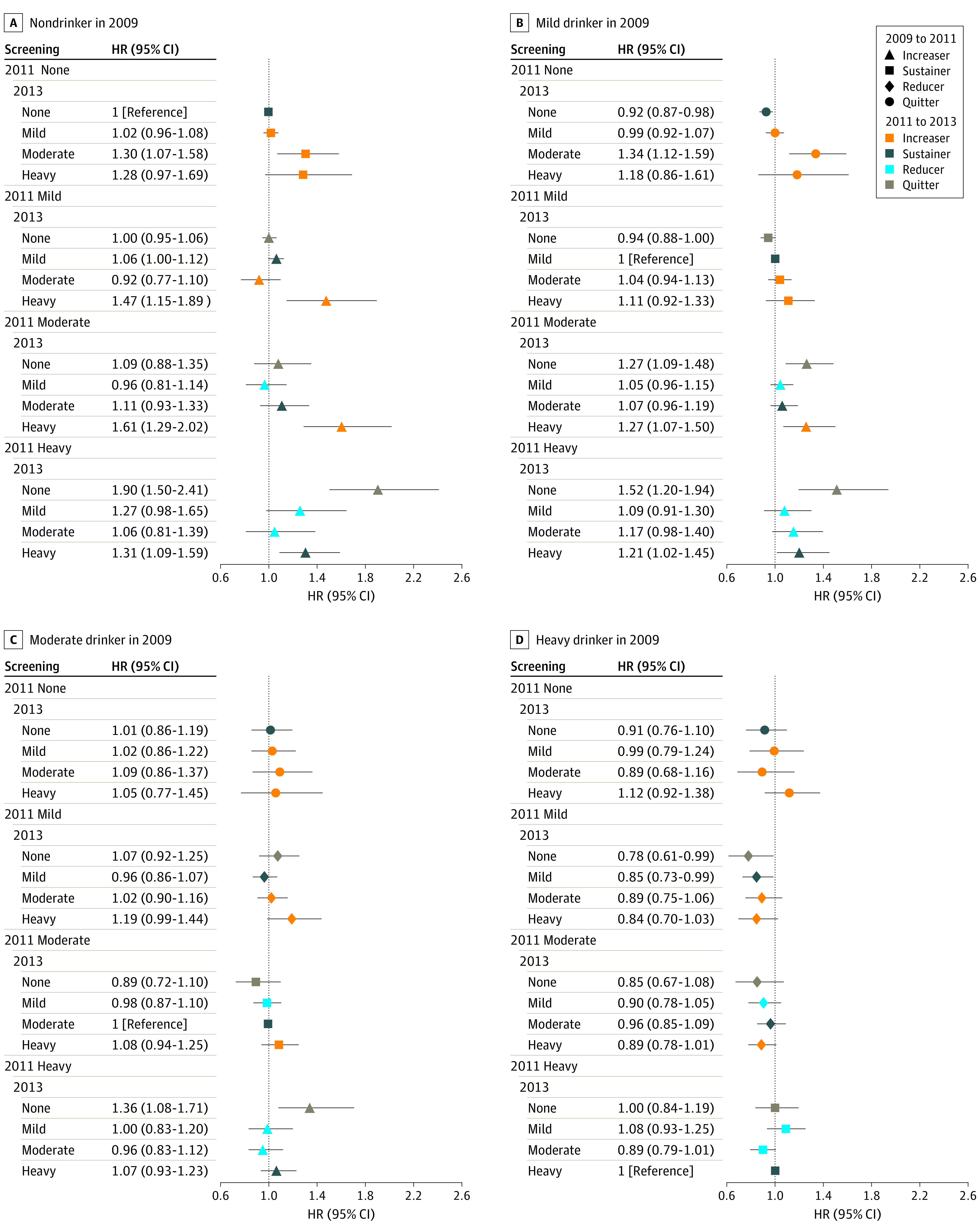
Risk of Alcohol-Related Cancers by Changes in Drinking Level From 2009 to 2013 Sustainers from 2009 to 2013 were the reference. Hazard ratios (HRs) were adjusted for age, sex, socioeconomic position (income level and place of residence), smoking status, physical activity, comorbidities, and Charlson Comorbidity Index. Error bars indicate the 95% CIs.

**Figure 3.**
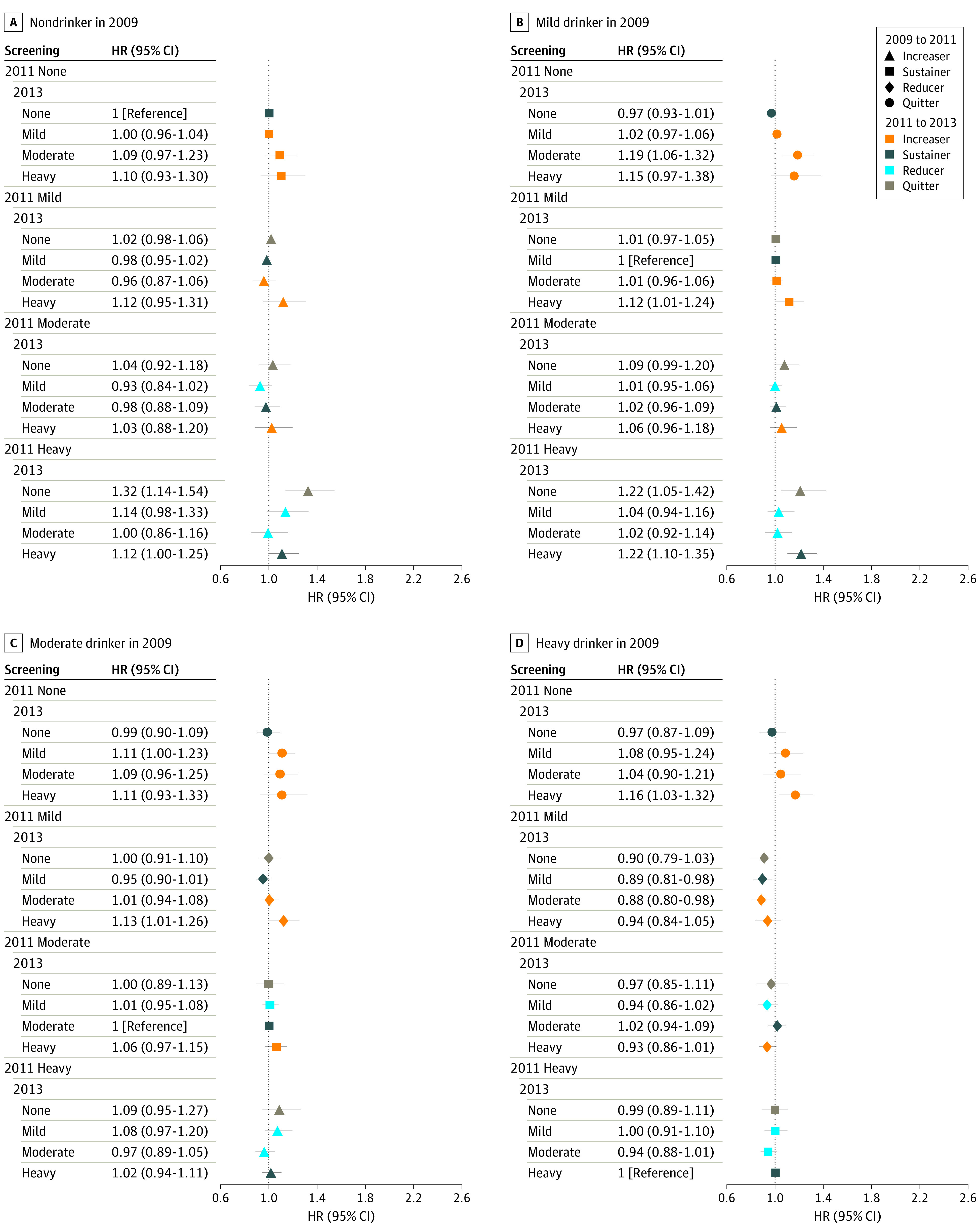
Risk of All Cancers by Changes in Drinking Level From 2009 to 2013 Sustainers from 2009 to 2013 were the reference. Hazard ratios (HRs) were adjusted for age, sex, socioeconomic position (income level and place of residence), smoking status, physical activity, comorbidities, and Charlson Comorbidity Index. Error bars indicate the 95% CIs.

We analyzed alcohol behavior from 3 consecutive health screenings for participants whose data were available (eTables 3 and 4 in the Supplement). Those who quit drinking at the 2011 screening and remained nondrinking at the 2013 screening no longer showed an increased incidence of alcohol-related cancers compared with those who sustained the same level of drinking from baseline: mild drinking (aHR, 0.92; 95% CI, 0.87-0.98), moderate drinking (aHR, 1.01; 95% CI, 0.86-1.19), and heavy drinking (aHR, 0.91; 95% CI, 0.76-1.10) (Figure 2). A similar pattern was observed for all cancers. We also found that those with heavy drinking levels who greatly reduced their alcohol intake to mild drinking levels at the 2011 screening and maintained mild drinking levels were found to have an even lower rate of alcohol-related cancers (aHR, 0.85; 95% CI, 0.73-0.99) (Figure 2). Those with heavy drinking levels who greatly decreased their alcohol consumption to mild drinking levels at the 2011 screening and maintained mild (aHR, 0.89; 95% CI, 0.81-0.98) to moderate (aHR, 0.88; 95% CI, 0.80-0.98) drinking levels were found to have an even lower rate of all cancers (Figure 3).

### Stratified Analysis

In stratified analyses according to age, sex, and smoking status, increased alcohol consumption was generally associated with an increased incidence of alcohol-related cancers and all cancers compared with sustained drinking, whereas a reduction in heavy drinking was generally associated with lower cancer risk, which was consistent with the main findings (eTables 5 to 8 in the Supplement). Associations between the change in alcohol consumption level and alcohol-related and all cancers were more prominent in participants who were older (eg, from nondrinking to heavy drinking in men aged ≥65 vs <65 years for alcohol-related cancers: aHR, 1.70 [95% CI, 1.48-1.94] vs 1.34 [95% CI, 1.20-1.50]), were male (eg, from nondrinking to heavy drinking in men vs women for alcohol-related cancers: aHR, 1.47 [95% CI, 1.35-1.60] vs 0.83 [95% CI, 0.60-1.16]), and had never smoker status (eg, from nondrinking to heavy drinking in never smoker vs current smoker with ≥20 pack-years for alcohol-related cancers: aHR, 1.77 [95% CI, 1.51-2.08] vs 1.49 [95% CI, 1.27-1.75]).

## Discussion

In this large cohort study that used repeated measurements of alcohol consumption, we found that individuals who increased their alcohol consumption, regardless of their baseline drinking level, had an increased incidence of alcohol-related and all cancers compared with those who sustained their current level of drinking. Quitting was not associated with a lower incidence of alcohol-related cancer, but if abstinence was maintained over time, the incidence of alcohol-related and all cancers tended to decrease. Reducing drinking from heavy to moderate or mild levels was associated with a decreased risk of alcohol-related and all cancers.

Consumption of alcoholic drinks is an established risk for so-called alcohol-related cancers, such as mouth, pharynx and larynx, esophagus, liver, colorectum, and breast cancers.^15,20^ In addition, those who increased their level of drinking had a higher risk of dose-associated alcohol-related cancers than those who sustained their level of drinking. Although we did not find this association consistently for all alcohol-related cancers except liver cancer (eTable 2 and eFigure 2 in the Supplement), the findings may support a potential causal association between alcohol consumption and cancers.

We found an elevated risk for all cancers among participants who recently quit drinking compared with those who sustained their level of drinking. An explanation for this phenomenon may be the *sick quitter phenomenon*,^21^ the idea that individuals could have stopped consuming alcohol after feeling symptoms and/or other adverse health effects. Although we conducted a primary analysis with 2 assessments and a 1-year lag, it was not enough to address the sick quitter bias.^22^ However, in subgroup analyses with people undertaking 3 measurements, participants who quit drinking by the 2011 screening and remained nondrinking at the 2013 screening showed similar or even decreased risk compared with those who sustained the same level of drinking.

Although the potential mechanism underlying alcohol-induced carcinogenesis is not fully understood, cases of alcohol-induced carcinogenesis might be reversible because of physiologic homeostasis after quitting drinking. For example, 4-fold to 10-fold increases in cytochrome P450 2E1 (CYP2E1) have been found to be associated with alcohol consumption,^23^ which result in the production of acetaldehyde, reactive oxygen species, and other carcinogenic substrates, such as nitrosamines,^24^ whereas a rapid decline in CYP2E1 expression has been reported 3 days after the cessation of alcohol consumption.^23^ In addition, numerous reports have observed an association between drinking alcohol and suppression of natural killer (NK) cell function.^25^ Alcohol consumption is a factor in inhibiting the effector function of NK cells, suppressing its cytolytic activity, blocking NK cell release, and inducing NK cell apoptosis.^25^ However, after 3 months of alcohol withdrawal, the number of NK cells continue to increase.^26^ Thereafter, the increased cancer risk from alcohol consumption can be reversible by the cessation of drinking, which is consistent with results of previous studies.^5,6,7,22,27,28^

In addition, we observed that the risks of alcohol-related and all cancers tended to decrease slightly for those with mild drinking levels who quit drinking. Previous studies have raised concerns that drinking even a small amount of alcohol increases the risk of cancer,^29^ including most upper aerodigestive tract cancers and gastrointestinal cancers.^30,31^ The present study highlights that there is no safe level of alcohol consumption in terms of cancer risk.

Although participants with a heavy drinking level—even those who reduced their alcohol intake later—still had a higher cancer risk than those with sustained nondrinking (eTable 9 in the Supplement), we observed that those who reduced their heavy drinking to a mild or moderate level had a decreased risk of cancer compared with those with sustained heavy drinking levels. There may be several explanations for this finding. First, alcohol consumed per se could have directly contributed to the decreased risk of cancer among the reducer group. Given the well-established dose-response association between the amount of alcohol consumption and several cancer risks,^32^ it is likely that reduction in alcohol consumption is a factor in lower risk of cancers. Second, reducing alcohol intake could act as a temporary step toward permanent quitting, and decreased cancer risk among the reducer group could be associated with their increased probability of complete abstinence.

When we analyzed the risk by sex, we found that the association between changes in drinking level and cancer risks were not significant for women. The lack of an observed association may be attributable to sample size constraints. Throughout the study period, 73.1% of female participants had a nondrinking level, and not enough women changed their drinking levels (eg, from heavy to mild or none) to show statistical significance. In addition, men generally engaged in binge drinking more than women. An association between alcohol consumption and cancer may be mediated by dose, duration, and pattern of alcohol intake, including binge drinking.^25^ In vivo studies in mice showed that binge alcohol exposure was associated with inhibited activity of NK cells and reduced number and lytic activity of NK cells.^25^ Because of these sex-related differences in drinking patterns, it is possible that the association of alcohol consumption with cancer risk was less prominent in women than in men. In line with the results of this study, the American Cancer Society^33^ and World Cancer Research Fund and American Institute for Cancer Research^4^ recommend not drinking alcohol at all given that the less alcohol consumed, the lower the risk of cancer.

### Limitations

This study has several limitations. First, we obtained lifestyle data, including level of alcohol consumption, from self-administered questionnaires, and it is probable that participants, especially women, underreported their alcohol consumption.^34,35^ Second, although examining long-term alcohol intake data before 2009 would be informative, information about long-term habits was not available from the NHIS database. The data covered a relatively short time span, preventing trajectory analyses or investigations of the impact of early-adulthood alcohol consumption.^36,37^ Third, because this study used data that were not originally designed for studying alcohol consumption, we were not able to assess aldehyde dehydrogenase gene status among participants, and we did not have pertinent information, such as reasons for reducing or stopping drinking and duration of drinking. Fourth, the results showing sex-based differences require cautious interpretation, which we attempted to address by performing sex-stratified analyses, because of the small number of women who reported drinking alcohol and even smaller number of women who reported heavy drinking levels. In addition, given the biological differences in alcohol metabolism between sexes, further studies that define drinking levels separately by sex may provide more insight into these associations among women.

Fifth, there might be unmeasured confounders, particularly those that would not be identified through routine health screening, such as stress or other mental health factors. Sixth, we did not consider concomitant health behavior changes in the analyses. It is possible that participants whose alcohol consumption behaviors changed over time may also experience changes in smoking status or physical activity. However, the sensitivity analysis among nonsmokers showed consistent results, suggesting that confounding from concomitant health behavior changes was not significant.

## Conclusions

This large, population-based cohort study found that an increased level of alcohol consumption was associated with higher incidence of alcohol-related cancers and all cancers compared with sustained drinking levels, whereas reduced drinking (particularly heavy drinking) was associated with a lower cancer risk. Quitting drinking was associated with increased risk, but this risk increase disappeared when quitting was sustained. Alcohol cessation and reduction should be reinforced for the prevention of cancer.

## Notes

Supplement. **eTable 1.** Associations Between Changes in Drinking Level and Cancer Including Participants With Previous Cardiovascular History **eTable 2.** The Risk of Site-Specific Cancer According to Changes in Drinking Level Between 2009 and 2011 **eTable 3.** Associations Between Changes in Drinking Level From 2009 to 2013 and Alcohol-Related Cancers **eTable 4.** Associations Between Changes in Drinking Level From 2009 to 2013 and All Cancers **eTable 5.** Adjusted Hazard Ratios and 95% Confidence Intervals for Cancer According to Changes in Drinking Level by Age **eTable 6.** Adjusted Hazard Ratios and 95% Confidence Intervals for Cancer According to Changes in Drinking Level by Sex **eTable 7.** Adjusted Hazard Ratios and 95% Confidence Intervals for Cancer According to Changes in Drinking Level by Smoking Status in 2009 (Male) **eTable 8.** Adjusted Hazard Ratios and 95% Confidence Intervals for Cancer According to Changes in Drinking Level by Smoking Status in 2009 (Female) **eTable 9.** Associations Between Changes in Drinking Level and Cancer (Sustained Non-Drinker as a Referent) **eFigure 1.** Flow Chart of the Study Population **eFigure 2.** The Risk of Site-Specific Cancer According to Changes in Drinking Level Between 2009 and 2011
