# Supplementary material for: Association Between Changes in Alcohol Consumption and Cancer Risk
Source: JAMA Netw Open. 2022 Aug 24;5(8):e2228544. doi: 10.1001/jamanetworkopen.2022.28544 (PMC9403779; doi:10.1001/jamanetworkopen.2022.28544)
Supplement: Supplement. — eTable 1. Associations Between Changes in Drinking Level and Cancer Including Participants With Previous Cardiovascular History eTable 2. The Risk of Site-Specific Cancer According to Changes in Drinking Level Between 2009 and 2011 eTable 3. Associations Between Changes in Drinking Level From 2009 to 2013 and Alcohol-Related Cancers eTable 4. Associations Between Changes in Drinking Level From 2009 to 2013 and All Cancers eTable 5. Adjusted Hazard Ratios and 95% Confidence Intervals for Cancer According to Changes in Drinking Level by Age eTable 6. Adjusted Hazard Ratios and 95% Confidence Intervals for Cancer According to Changes in Drinking Level by Sex eTable 7. Adjusted Hazard Ratios and 95% Confidence Intervals for Cancer According to Changes in Drinking Level by Smoking Status in 2009 (Male) eTable 8. Adjusted Hazard Ratios and 95% Confidence Intervals for Cancer According to Changes in Drinking Level by Smoking Status in 2009 (Female) eTable 9. Associations Between Changes in Drinking Level and Cancer (Sustained Non-Drinker as a Referent) eFigure 1. Flow Chart of the Study Population eFigure 2. The Risk of Site-Specific Cancer According to Changes in Drinking Level Between 2009 and 2011 [file jamanetwopen-e2228544-s001.pdf]

Supplemental Online Content

Yoo JE, Han K, Shin DW, et al. Association between changes in alcohol consumption and cancer risk. *JAMA Netw Open*. 2022;5(8):e2228544. doi:10.1001/jamanetworkopen.2022.28544

eTable 1. Associations Between Changes in Drinking Level and Cancer Including Participants With Previous Cardiovascular History

eTable 2. The Risk of Site-Specific Cancer According to Changes in Drinking Level Between 2009 and 2011

eTable 3. Associations Between Changes in Drinking Level From 2009 to 2013 and Alcohol-Related Cancers

eTable 4. Associations Between Changes in Drinking Level From 2009 to 2013 and All Cancers

eTable 5. Adjusted Hazard Ratios and 95% Confidence Intervals for Cancer According to Changes in Drinking Level by Age

eTable 6. Adjusted Hazard Ratios and 95% Confidence Intervals for Cancer According to Changes in Drinking Level by Sex

eTable 7. Adjusted Hazard Ratios and 95% Confidence Intervals for Cancer According to Changes in Drinking Level by Smoking Status in 2009 (Male)

eTable 8. Adjusted Hazard Ratios and 95% Confidence Intervals for Cancer According to Changes in Drinking Level by Smoking Status in 2009 (Female)

eTable 9. Associations Between Changes in Drinking Level and Cancer (Sustained Non-Drinker as a Referent)

eFigure 1. Flow Chart of the Study Population

eFigure 2. The Risk of Site-Specific Cancer According to Changes in Drinking Level Between 2009 and 2011

This supplemental material has been provided by the authors to give readers additional information about their work.

© 2022 Yoo JE et al. *JAMA Network Open.*

eTable 1. Associations Between Changes in Drinking Level and Cancer Including Participants With Previous Cardiovascular History

Alcohol consumption status		Subjects (N)	Events (n)	Person-years	IR	aHR (95% CI)*
**2009**	**2011**					
**Alcohol-related cancers**						
None	None	2,258,335	40,732	14,220,119.5	2.9	1 (Ref.)
	Mild	300,068	5,046	1,889,920.3	2.7	1.03 (1.00–1.06)
	Moderate	43,783	846	274,477.1	3.1	**1.11 (1.03–1.19)**
	Heavy	25,169	590	156,537.6	3.8	**1.33 (1.22–1.44)**
Mild	None	309,925	5,559	1,948,075.8	2.9	**0.96 (0.93–0.99)**
	Mild	666,087	10,347	4,195,312.0	2.5	1 (Ref.)
	Moderate	138,812	2,393	872,245.7	2.7	**1.09 (1.05–1.14)**
	Heavy	42,271	875	264,233.9	3.3	**1.17 (1.09–1.25)**
Moderate	None	45,507	1,015	283,064.5	3.6	1.04 (0.97–1.12)
	Mild	161,854	2,856	1,015,160.5	2.8	0.98 (0.93–1.03)
	Moderate	177,863	2,970	1,116,637.9	2.7	1 (Ref.)
	Heavy	80,059	1,512	501,377.0	3.0	1.04 (0.97–1.10)
Heavy	None	28,375	811	174,053.8	4.7	1.02 (0.95–1.11)
	Mild	53,992	1,165	336,554.5	3.5	**0.91 (0.85–0.97)**
	Moderate	89,022	1,757	556,166.9	3.2	**0.91 (0.86–0.97)**
	Heavy	150,971	3,428	941,527.4	3.6	1 (Ref.)
**All cancers (except thyroid)**						
None	None	2,258,335	108,031	14,063,390.2	7.7	1 (Ref.)
	Mild	300,068	13,251	1,870,327.8	7.1	0.99 (0.97–1.00)
	Moderate	43,783	2,356	270,848.9	8.7	0.99 (0.95–1.03)
	Heavy	25,169	1,642	154,197.0	10.6	**1.11 (1.06–1.17)**
Mild	None	309,925	15,088	1,925,700.3	7.8	0.98 (0.96–1.00)
	Mild	666,087	28,991	4,150,056.0	7.0	1 (Ref.)
	Moderate	138,812	6,616	862,319.3	7.7	1.02 (0.99–1.05)
	Heavy	42,271	2,428	260,607.7	9.3	**1.08 (1.04–1.13)**
Moderate	None	45,507	2,970	278,591.3	10.7	**1.07 (1.02–1.11)**
	Mild	161,854	8,045	1,002,858.3	8.0	0.98 (0.95–1.01)
	Moderate	177,863	8,305	1,104,090.9	7.5	1 (Ref.)
	Heavy	80,059	4,153	495,461.6	8.4	1.01 (0.97–1.05)
Heavy	None	28,375	2,257	171,028.4	13.2	**1.06 (1.01–1.11)**
	Mild	53,992	3,190	331,896.5	9.6	**0.92 (0.89–0.96)**
	Moderate	89,022	4,866	548,961.7	8.9	**0.95 (0.92–0.99)**
	Heavy	150,971	9,224	928,313.8	9.9	1 (Ref.)

IR, incidence rate per 1,000 person-years; aHR, adjusted hazard ratio; CI, confidence interval

*adjusted for age, sex, socioeconomic position (income level and place of residence), smoking status, physical activity, comorbidities (hypertension, diabetes, dyslipidemia, chronic kidney disease, and chronic obstructive pulmonary disease), and Charlson comorbidity index

© 2022 Yoo JE et al. *JAMA Network Open.*

eTable 2. The Risk of Site-Specific Cancer According to Changes in Drinking Level Between 2009 and 2011

2011 / 2009	None	Mild	Moderate	Heavy
**Lip, oral cavity and pharynx (n=3884, IR=0.14)**				
None	1 (Ref.)	0.94 (0.80–1.11)	1.00 (0.74–1.35)	1.13 (0.83–1.55)
Mild	**1.20 (1.04–1.38)**	1 (Ref.)	0.98 (0.81–1.19)	1.11 (0.87–1.42)
Moderate	1.21 (0.90–1.63)	1.15 (0.96–1.38)	1 (Ref.)	0.96 (0.77–1.20)
Heavy	**1.47 (1.06–2.05)**	1.22 (0.92–1.61)	0.93 (0.73–1.18)	1 (Ref.)
**Esophagus (n=3,009, IR=0.11)**				
None	1 (Ref.)	0.92 (0.76–1.12)	1.07 (0.81–1.41)	1.01 (0.79–1.29)
Mild	1.13 (0.92–1.38)	1 (Ref.)	**0.80 (0.66–0.98)**	**0.74 (0.59–0.92)**
Moderate	**2.38 (1.79–3.17)**	**1.38 (1.13–1.70)**	1 (Ref.)	**0.73 (0.61–0.88)**
Heavy	**3.66 (2.77–4.83)**	**2.23 (1.74–2.86)**	1.03 (0.83–1.29)	1 (Ref.)
**Stomach (n=38,225, IR=1.34)**				
None	1 (Ref.)	0.97 (0.92–1.02)	1.09 (0.98–1.20)	**1.12 (1.01–1.25)**
Mild	0.98 (0.94–1.03)	1 (Ref.)	0.99 (0.92–1.06)	0.92 (0.84–1.01)
Moderate	1.02 (0.93–1.13)	1.04 (0.98–1.11)	1 (Ref.)	0.96 (0.89–1.04)
Heavy	**1.16 (1.04–1.30)**	**1.16 (1.06–1.27)**	0.97 (0.90–1.06)	1 (Ref.)
**Colorectum (n=41,102, IR=1.44)**				
None	1 (Ref.)	0.96 (0.92–1.01)	1.01 (0.92–1.12)	1.02 (0.92–1.14)
Mild	**1.08 (1.03–1.12)**	1 (Ref.)	0.97 (0.91–1.04)	0.94 (0.86–1.03)
Moderate	1.09 (0.99–1.20)	**1.12 (1.06–1.19)**	1 (Ref.)	0.96 (0.89–1.04)
Heavy	**1.31 (1.17–1.46)**	**1.12 (1.03–1.23)**	1.04 (0.96–1.13)	1 (Ref.)
**Liver (n=15,333, IR=0.54)**				
None	1 (Ref.)	**1.12 (1.04–1.21)**	**1.26 (1.09–1.46)**	1.15 (1.00–1.33)
Mild	0.99 (0.92–1.06)	1 (Ref.)	1.03 (0.93–1.14)	0.91 (0.80–1.03)
Moderate	**1.25 (1.10–1.43)**	**1.10 (1.00–1.21)**	1 (Ref.)	**0.89 (0.80–0.99)**
Heavy	**1.39 (1.20–1.62)**	1.11 (0.96–1.28)	1.11 (0.99–1.26)	1 (Ref.)
**Gallbladder (n=9,244, IR=0.32)**				
None	1 (Ref.)	**0.89 (0.80–0.99)**	**1.28 (1.04–1.57)**	**1.29 (1.03–1.61)**
Mild	0.98 (0.89–1.07)	1 (Ref.)	0.92 (0.78–1.08)	0.86 (0.70–1.07)
Moderate	**1.25 (1.02–1.53)**	1.06 (0.93–1.22)	1 (Ref.)	1.04 (0.87–1.24)
Heavy	0.95 (0.72–1.26)	1.16 (0.95–1.42)	1.07 (0.89–1.29)	1 (Ref.)
**Pancreas (n=15,691, IR=0.55)**				
None	1 (Ref.)	1.04 (0.97–1.12)	1.02 (0.87–1.19)	1.06 (0.90–1.25)
Mild	1.01 (0.95–1.08)	1 (Ref.)	1.04 (0.94–1.16)	0.92 (0.80–1.06)
Moderate	0.89 (0.76–1.04)	1.07 (0.97–1.17)	1 (Ref.)	0.93 (0.83–1.05)
Heavy	1.15 (0.97–1.36)	0.96 (0.83–1.11)	1.02 (0.90–1.15)	1 (Ref.)
**Larynx (n=1,642, IR=0.55)**				
None	1 (Ref.)	1.01 (0.79–1.29)	1.10 (0.76–1.60)	1.31 (0.88–1.95)
Mild	1.10 (0.86–1.41)	1 (Ref.)	**0.73 (0.55–0.95)**	1.10 (0.80–1.53)
Moderate	**1.65 (1.12–2.41)**	1.11 (0.85–1.45)	1 (Ref.)	0.93 (0.69–1.24)
Heavy	1.51 (0.95–2.41)	**2.10 (1.55–2.85)**	0.75 (0.54–1.03)	1 (Ref.)
**Lung (n=32,096, IR=1.12)**				
None	1 (Ref.)	**1.08 (1.02–1.14)**	**1.17 (1.05–1.31)**	**1.15 (1.02–1.28)**
Mild	**0.91 (0.86–0.96)**	1 (Ref.)	1.01 (0.93–1.09)	0.96 (0.87–1.06)
Moderate	**0.87 (0.78–0.97)**	1.02 (0.95–1.09)	1 (Ref.)	1.00 (0.92–1.09)
Heavy	1.03 (0.91–1.15)	1.00 (0.90–1.11)	0.98 (0.89–1.08)	1 (Ref.)
**Breast (n=20,532, IR=0.72)**				
None	1 (Ref.)	0.97 (0.91–1.04)	0.83 (0.63–1.10)	1.07 (0.69–1.67)
Mild	**0.94 (0.88–0.99)**	1 (Ref.)	0.83 (0.66–1.03)	0.69 (0.45–1.06)

© 2022 Yoo JE et al. *JAMA Network Open.*

Moderate	0.92 (0.74–1.16)	1.02 (0.87–1.19)	1 (Ref.)	1.15 (0.77–1.71)
Heavy	0.76 (0.49–1.17)	0.95 (0.68–1.32)	**0.64 (0.43–0.97)**	1 (Ref.)
**Cervix uteri (n=3,048, IR=0.11)**				
None	1 (Ref.)	0.93 (0.78–1.10)	1.10 (0.53–2.28)	1.36 (0.46–4.06)
Mild	**1.23 (1.08–1.41)**	1 (Ref.)	0.84 (0.44–1.59)	1.51 (0.56–4.07)
Moderate	0.99 (0.55–1.79)	0.77 (0.50–1.20)	1 (Ref.)	1.30 (0.45–3.77)
Heavy	1.16 (0.43–3.10)	1.15 (0.54–2.43)	1.04 (0.38–2.86)	1 (Ref.)
**Corpus uteri (n=2,828, IR=0.10)**				
None	1 (Ref.)	0.98 (0.81–1.18)	1.81 (0.63–5.19)	6.34 (0.75–53.77)
Mild	**0.85 (0.73–0.99)**	1 (Ref.)	1.74 (0.70–4.36)	6.63 (0.84–52.31)
Moderate	1.15 (0.67–1.99)	0.80 (0.49–1.31)	1 (Ref.)	3.57 (0.40–32.00)
Heavy	0.89 (0.29–2.77)	1.36 (0.61–3.07)	**4.21 (1.46–12.18)**	1 (Ref.)
**Ovary (n=3,866, IR=0.14)**				
None	1 (Ref.)	0.97 (0.84–1.13)	0.86 (0.44–1.66)	0.68 (0.24–1.94)
Mild	1.03 (0.92–1.16)	1 (Ref.)	**0.51 (0.28–0.92)**	1.16 (0.52–2.59)
Moderate	1.06 (0.67–1.68)	0.81 (0.56–1.19)	1 (Ref.)	0.55 (0.20–1.53)
Heavy	0.95 (0.39–2.28)	0.78 (0.35–1.74)	0.93 (0.40–2.18)	1 (Ref.)
**Prostate (n=20,990, IR=0.73)**				
None	1 (Ref.)	0.97 (0.91–1.02)	1.01 (0.90–1.13)	0.95 (0.83–1.08)
Mild	1.04 (0.99–1.10)	1 (Ref.)	0.94 (0.87–1.02)	0.94 (0.84–1.04)
Moderate	1.08 (0.97–1.20)	0.93 (0.87–1.00)	1 (Ref.)	0.98 (0.90–1.08)
Heavy	**1.15 (1.01–1.30)**	0.93 (0.83–1.04)	1.01 (0.92–1.12)	1 (Ref.)
**Kidney (n=7,771, IR=0.27)**				
None	1 (Ref.)	1.00 (0.90–1.12)	0.83 (0.65–1.05)	0.96 (0.74–1.24)
Mild	0.96 (0.87–1.06)	1 (Ref.)	**0.85 (0.73–0.99)**	0.85 (0.69–1.04)
Moderate	0.97 (0.78–1.21)	1.02 (0.89–1.17)	1 (Ref.)	0.99 (0.84–1.17)
Heavy	1.10 (0.86–1.42)	0.89 (0.71–1.12)	**0.73 (0.60–0.89)**	1 (Ref.)
**Bladder (n=7,893, IR=0.28)**				
None	1 (Ref.)	0.98 (0.88–1.08)	0.95 (0.77–1.19)	1.07 (0.85–1.35)
Mild	1.01 (0.92–1.12)	1 (Ref.)	**0.82 (0.70–0.95)**	**0.75 (0.61–0.93)**
Moderate	0.96 (0.78–1.18)	0.91 (0.80–1.05)	1 (Ref.)	0.97 (0.82–1.14)
Heavy	1.22 (0.98–1.52)	0.93 (0.76–1.14)	**0.81 (0.67–0.97)**	1 (Ref.)
**Brain and central nerve system (n=3,613, IR=0.13)**				
None	1 (Ref.)	**1.18 (1.00–1.39)**	1.03 (0.71–1.49)	1.38 (0.92–2.07)
Mild	**0.84 (0.72–0.98)**	1 (Ref.)	1.22 (0.96–1.55)	1.16 (0.83–1.63)
Moderate	1.12 (0.82–1.52)	1.12 (0.90–1.38)	1 (Ref.)	1.05 (0.78–1.41)
Heavy	1.38 (0.97–1.96)	1.08 (0.76–1.52)	0.86 (0.62–1.19)	1 (Ref.)
**Hodgkin lymphoma (n=309, IR=0.01)**				
None	1 (Ref.)	0.76 (0.40–1.44)	N/A	1.19 (0.32–4.45)
Mild	0.60 (0.34–1.05)	1 (Ref.)	1.15 (0.53–2.47)	1.20 (0.42–3.46)
Moderate	0.85 (0.31–2.33)	0.88 (0.42–1.81)	1 (Ref.)	0.75 (0.26–2.12)
Heavy	0.33 (0.05–2.38)	1.45 (0.57–3.70)	0.95 (0.36–2.51)	1 (Ref.)
**Non-Hodgkin lymphoma (n=5,558, IR=0.19)**				
None	1 (Ref.)	0.96 (0.84–1.10)	1.05 (0.78–1.41)	1.10 (0.80–1.51)
Mild	**0.84 (0.74–0.95)**	1 (Ref.)	1.00 (0.82–1.23)	**0.73 (0.54–0.98)**
Moderate	0.79 (0.59–1.06)	1.08 (0.92–1.28)	1 (Ref.)	0.94 (0.75–1.18)
Heavy	**0.64 (0.42–0.96)**	1.06 (0.81–1.39)	0.80 (0.62–1.04)	1 (Ref.)
**Multiple myeloma (n=2,353, IR=0.08)**				
None	1 (Ref.)	1.03 (0.85–1.25)	1.43 (0.93–2.18)	**1.86 (1.22–2.84)**
Mild	0.93 (0.78–1.10)	1 (Ref.)	1.11 (0.81–1.53)	0.65 (0.39–1.07)
Moderate	1.22 (0.86–1.74)	0.88 (0.68–1.16)	1 (Ref.)	1.05 (0.73–1.51)
Heavy	0.89 (0.53–1.49)	0.70 (0.44–1.14)	0.93 (0.62–1.39)	1 (Ref.)
**Leukemia (n=3,533, IR=0.12)**				

© 2022 Yoo JE et al. *JAMA Network Open.*

None	1 (Ref.)	**1.25 (1.06–1.47)**	1.08 (0.77–1.51)	1.10 (0.76–1.59)
Mild	0.89 (0.76–1.04)	1 (Ref.)	0.91 (0.72–1.16)	0.77 (0.55–1.09)
Moderate	1.03 (0.74–1.44)	**1.26 (1.02–1.54)**	1 (Ref.)	0.78 (0.58–1.05)
Heavy	1.03 (0.68–1.56)	1.27 (0.92–1.77)	0.83 (0.61–1.13)	1 (Ref.)

IR, incidence rate per 1,000 person-years
HRs are adjusted for age, sex, socioeconomic position (income level and place of residence), smoking status, physical activity, comorbidities (hypertension, diabetes, dyslipidemia, chronic kidney disease, and chronic obstructive pulmonary disease), and Charlson comorbidity index

© 2022 Yoo JE et al. *JAMA Network Open.*

eTable 3. Associations Between Changes in Drinking Level From 2009 to 2013 and Alcohol-Related Cancers

Alcohol consumption status			Subject	Event	IR	aHR (95% CI)*
2009	2011	2013				
None	None	None	19,873	7,087,021.8	2.8	1 (Ref.)
		Mild	1,195	459,964.8	2.6	1.02 (0.96–1.08)
		Moderate	114	31,792.7	3.6	**1.30 (1.07–1.58)**
		Heavy	49	15,031.9	3.3	1.28 (0.97–1.69)
	Mild	None	1,223	465,526.9	2.6	1.00 (0.95–1.06)
		Mild	1,232	484,216.0	2.5	1.06 (1.00–1.12)
		Moderate	133	55,021.4	2.4	0.92 (0.77–1.10)
		Heavy	62	16,407.7	3.8	**1.47 (1.15–1.89)**
	Moderate	None	86	27,945.3	3.1	1.09 (0.88–1.35)
		Mild	148	54,827.5	2.7	0.96 (0.81–1.14)
		Moderate	122	43,820.8	2.8	1.11 (0.93–1.33)
		Heavy	73	19,198.6	3.8	**1.61 (1.29–2.02)**
	Heavy	None	70	12,669.7	5.5	**1.90 (1.50–2.41)**
		Mild	52	17,250.3	3.0	1.27 (0.98–1.65)
		Moderate	51	19,761.5	2.6	1.06 (0.81–1.39)
		Heavy	112	31,997.6	3.5	**1.31 (1.09–1.59)**
Mild	None	None	1,801	630,737.2	2.9	**0.92 (0.87–0.98)**
		Mild	941	365,885.6	2.6	0.99 (0.92–1.07)
		Moderate	135	38,276.9	3.5	**1.34 (1.12–1.59)**
		Heavy	45	13,242.9	3.4	1.18 (0.86–1.61)
	Mild	None	1,208	469,311.3	2.6	0.94 (0.88–1.00)
		Mild	3,639	1,580,043.6	2.3	1 (Ref.)
		Moderate	540	225,312.6	2.4	1.04 (0.94–1.13)
		Heavy	126	44,868.2	2.8	1.11 (0.92–1.33)
	Moderate	None	174	45,075.0	3.9	**1.27 (1.09–1.48)**
		Mild	562	225,463.1	2.5	1.05 (0.96–1.15)
		Moderate	405	161,040.8	2.5	1.07 (0.96–1.19)
		Heavy	127	45,993.6	2.8	**1.27 (1.07–1.50)**
	Heavy	None	64	14,504.8	4.4	**1.52 (1.20–1.94)**
		Mild	133	43,585.5	3.1	1.09 (0.91–1.30)
		Moderate	129	42,848.4	3.0	1.17 (0.98–1.40)
		Heavy	132	39,853.1	3.3	**1.21 (1.02–1.45)**
Moderate	None	None	203	52,042.1	3.9	1.01 (0.86–1.19)
		Mild	162	52,099.8	3.1	1.02 (0.86–1.22)
		Moderate	82	29,913.8	2.7	1.09 (0.86–1.37)
		Heavy	49	14,015.8	3.5	1.05 (0.77–1.45)
	Mild	None	206	59,528.0	3.5	1.07 (0.92–1.25)
		Mild	786	301,074.6	2.6	0.96 (0.86–1.07)
		Moderate	404	157,258.8	2.6	1.02 (0.90–1.16)
		Heavy	139	40,680.5	3.4	1.19 (0.99–1.44)
	Moderate	None	106	35,153.2	3.0	0.89 (0.72–1.10)
		Mild	486	189,577.0	2.6	0.98 (0.87–1.10)
		Moderate	694	290,797.5	2.4	1 (Ref.)
		Heavy	275	100,466.1	2.7	1.08 (0.94–1.25)
	Heavy	None	83	16,720.4	5.0	**1.36 (1.08–1.71)**
		Mild	143	48,011.7	3.0	1.00 (0.83–1.20)

© 2022 Yoo JE et al. *JAMA Network Open.*

		Moderate	255	100,260.7	2.5	0.96 (0.83–1.12)
		Heavy	299	105,201.9	2.8	1.07 (0.93–1.23)
Heavy	None	None	134	28,090.3	4.8	0.91 (0.76–1.10)
		Mild	80	19,988.6	4.0	0.99 (0.79–1.24)
		Moderate	68	17,076.1	4.0	0.89 (0.68–1.16)
		Heavy	106	23,577.0	4.5	1.12 (0.92–1.38)
	Mild	None	79	20,587.8	3.8	**0.78 (0.61–0.99)**
		Mild	213	70,216.5	3.0	**0.85 (0.73–0.99)**
		Moderate	159	51,763.6	3.1	0.89 (0.75–1.06)
		Heavy	120	36,426.5	3.3	0.84 (0.70–1.03)
	Moderate	None	77	18,980.9	4.1	0.85 (0.67–1.08)
		Mild	202	65,248.8	3.1	0.90 (0.78–1.05)
		Moderate	380	119,628.3	3.2	0.96 (0.85–1.09)
		Heavy	289	96,593.4	3.0	0.89 (0.78–1.01)
	Heavy	None	138	28,663.2	4.8	1.00 (0.84–1.19)
		Mild	222	51,153.0	4.3	1.08 (0.93–1.25)
		Moderate	362	116,944.3	3.1	0.89 (0.79–1.01)
		Heavy	1,075	304,657.6	3.5	1 (Ref.)

IR, incidence rate per 1,000 person-years; aHR, adjusted hazard ratio; CI, confidence interval
*Adjusted for age, sex, socioeconomic position (income level and place of residence), smoking status, physical activity, comorbidities (hypertension, diabetes, dyslipidemia, chronic kidney disease, and chronic obstructive pulmonary disease), and Charlson comorbidity index

© 2022 Yoo JE et al. *JAMA Network Open.*

eTable 4. Associations Between Changes in Drinking Level From 2009 to 2013 and All Cancers

Alcohol consumption status			Subject	Event	IR	aHR (95% CI)*
2009	2011	2013				
None	None	None	1,614,929	54,098	7.7	1 (Ref.)
		Mild	105,266	3,143	6.9	1.00 (0.96–1.04)
		Moderate	7,323	299	9.5	1.09 (0.97–1.23)
		Heavy	3,455	160	10.8	1.10 (0.93–1.30)
	Mild	None	106,383	3,402	7.4	1.02 (0.98–1.06)
		Mild	110,885	3,311	6.9	0.98 (0.95–1.02)
		Moderate	12,608	421	7.7	0.96 (0.87–1.06)
		Heavy	3,758	161	9.9	1.12 (0.95–1.31)
	Moderate	None	6,394	270	9.8	1.04 (0.92–1.18)
		Mild	12,551	443	8.2	0.93 (0.84–1.02)
		Moderate	10,053	357	8.2	0.98 (0.88–1.09)
		Heavy	4,408	179	9.4	1.03 (0.88–1.20)
	Heavy	None	2,922	172	13.7	**1.32 (1.14–1.54)**
		Mild	3,961	170	10.0	1.14 (0.98–1.33)
		Moderate	4,545	180	9.2	1.00 (0.86–1.16)
		Heavy	7,354	342	10.8	**1.12 (1.00–1.25)**
Mild	None	None	144,117	5,054	8.1	0.97 (0.93–1.01)
		Mild	83,735	2,651	7.3	1.02 (0.97–1.06)
		Moderate	8,773	358	9.4	**1.19 (1.06–1.32)**
		Heavy	3,037	132	10.1	1.15 (0.97–1.38)
	Mild	None	107,498	3,680	7.9	1.01 (0.97–1.05)
		Mild	362,504	10,606	6.8	1 (Ref.)
		Moderate	51,708	1,627	7.3	1.01 (0.96–1.06)
		Heavy	10,288	386	8.7	**1.12 (1.01–1.24)**
	Moderate	None	10,340	463	10.4	1.09 (0.99–1.20)
		Mild	51,765	1,675	7.5	1.01 (0.95–1.06)
		Moderate	36,959	1,181	7.4	1.02 (0.96–1.09)
		Heavy	10,567	369	8.1	1.06 (0.96–1.18)
	Heavy	None	3,341	178	12.4	**1.22 (1.05–1.42)**
		Mild	10,012	382	8.9	1.04 (0.94–1.16)
		Moderate	9,840	355	8.4	1.02 (0.92–1.14)
		Heavy	9,158	418	10.6	**1.22 (1.10–1.35)**
Moderate	None	None	11,942	603	11.7	0.99 (0.90–1.09)
		Mild	11,964	522	10.1	**1.11 (1.00–1.23)**
		Moderate	6,861	270	9.1	1.09 (0.96–1.25)
		Heavy	3,215	142	10.2	1.11 (0.93–1.33)
	Mild	None	13,689	611	10.4	1.00 (0.91–1.10)
		Mild	69,131	2,286	7.7	0.95 (0.90–1.01)
		Moderate	36,098	1,195	7.7	1.01 (0.94–1.08)
		Heavy	9,362	400	9.9	**1.13 (1.01–1.26)**
	Moderate	None	8,070	367	10.6	1.00 (0.89–1.13)
		Mild	43,605	1,523	8.1	1.01 (0.95–1.08)
		Moderate	66,854	2,073	7.2	1 (Ref.)
		Heavy	23,092	801	8.0	1.06 (0.97–1.15)
	Heavy	None	3,876	217	13.2	1.09 (0.95–1.27)
		Mild	11,030	457	9.6	1.08 (0.97–1.20)

© 2022 Yoo JE et al. *JAMA Network Open.*

		Moderate	23,066	783	7.9	0.97 (0.89–1.05)
		Heavy	24,184	874	8.4	1.02 (0.94–1.11)
Heavy	None	None	6,515	398	14.4	0.97 (0.87–1.09)
		Mild	4,605	250	12.7	1.08 (0.95–1.24)
		Moderate	3,937	190	11.3	1.04 (0.90–1.21)
		Heavy	5,418	302	13.0	**1.16 (1.03–1.32)**
	Mild	None	4,726	256	12.6	0.90 (0.79–1.03)
		Mild	16,139	620	8.9	**0.89 (0.81–0.98)**
		Moderate	11,911	440	8.6	**0.88 (0.80–0.98)**
		Heavy	8,369	362	10.0	0.94 (0.84–1.05)
	Moderate	None	4,379	237	12.7	0.97 (0.85–1.11)
		Mild	15,049	607	9.4	0.94 (0.86–1.02)
		Moderate	27,597	1,054	8.9	1.02 (0.94–1.09)
		Heavy	22,232	825	8.6	0.93 (0.86–1.01)
	Heavy	None	6,616	411	14.6	0.99 (0.89–1.11)
		Mild	11,822	569	11.2	1.00 (0.91–1.10)
		Moderate	26,960	1,054	9.1	0.94 (0.88–1.01)
		Heavy	70,176	2,983	9.9	1 (Ref.)

IR, incidence rate per 1,000 person-years; aHR, adjusted hazard ratio; CI, confidence interval
*Adjusted for age, sex, socioeconomic position (income level and place of residence), smoking status, physical activity, comorbidities (hypertension, diabetes, dyslipidemia, chronic kidney disease, and chronic obstructive pulmonary disease), and Charlson comorbidity index

eTable 5. Adjusted Hazard Ratios* and 95% Confidence Intervals for Cancer According to Changes in Drinking Level by Age

2009 \ 2011		Male				Female			
		**None**	**Mild**	**Moderate**	**Heavy**	**None**	**Mild**	**Moderate**	**Heavy**
**Alcohol-related cancers**									
<65 years	None	1 (Ref.)	1.05 (1.00–1.12)	**1.14 (1.03–1.26)**	**1.34 (1.20–1.50)**	1 (Ref.)	0.99 (0.94–1.03)	0.96 (0.80–1.15)	0.85 (0.60–1.21)
	Mild	**1.07 (1.01–1.13)**	1 (Ref.)	**1.10 (1.04–1.17)**	**1.20 (1.09–1.31)**	0.99 (0.94–1.05)	1 (Ref.)	1.04 (0.91–1.18)	0.81 (0.60–1.09)
	Moderate	**1.12 (1.01–1.24)**	1.02 (0.96–1.09)	1 (Ref.)	1.04 (0.96–1.12)	0.92 (0.73–1.15)	0.81 (0.67–0.98)	1 (Ref.)	0.68 (0.49–0.96)
	Heavy	**1.13 (1.01–1.26)**	0.94 (0.86–1.03)	**0.91 (0.85–0.98)**	1 (Ref.)	0.95 (0.64–1.41)	0.87 (0.61–1.23)	1.11 (0.79–1.57)	1 (Ref.)
≥65 years	None	1 (Ref.)	**1.11 (1.04–1.19)**	**1.40 (1.23–1.59)**	**1.70 (1.48–1.94)**	1 (Ref.)	1.06 (0.95–1.18)	0.93 (0.57–1.52)	0.71 (0.27–1.89)
	Mild	0.98 (0.91–1.05)	1 (Ref.)	**1.21 (1.11–1.32)**	**1.21 (1.07–1.37)**	0.95 (0.81–1.12)	1 (Ref.)	0.97 (0.60–1.57)	1.26 (0.59–2.67)
	Moderate	1.10 (0.97–1.25)	0.91 (0.82–1.01)	1 (Ref.)	1.12 (0.99–1.26)	0.62 (0.27–1.42)	1.44 (0.69–3.03)	1 (Ref.)	1.36 (0.45–4.08)
	Heavy	1.00 (0.89–1.13)	**0.89 (0.79–0.99)**	**0.90 (0.81–1.00)**	1 (Ref.)	0.54 (0.16–1.81)	0.89 (0.29–2.76)	0.66 (0.16–2.78)	1 (Ref.)
**All cancers (except thyroid cancers)**									
<65 years	None	1 (Ref.)	1.00 (0.97–1.03)	1.04 (0.98–1.10)	**1.17 (1.10–1.26)**	1 (Ref.)	1.00 (0.96–1.03)	0.95 (0.83–1.09)	1.00 (0.80–1.27)
	Mild	**1.05 (1.01–1.08)**	1 (Ref.)	**1.04 (1.01–1.08)**	**1.11 (1.05–1.18)**	1.01 (0.97–1.06)	1 (Ref.)	1.03 (0.94–1.14)	1.00 (0.82–1.21)
	Moderate	**1.16 (1.09–1.24)**	0.99 (0.96–1.03)	1 (Ref.)	1.00 (0.96–1.05)	1.03 (0.87–1.22)	0.98 (0.85–1.13)	1 (Ref.)	0.85 (0.66–1.08)
	Heavy	**1.11 (1.04–1.19)**	0.94 (0.89–1.00)	0.96 (0.92–1.00)	1 (Ref.)	0.96 (0.72–1.29)	0.96 (0.74–1.24)	1.04 (0.80–1.36)	1 (Ref.)
≥65 years	None	1 (Ref.)	1.00 (0.96–1.04)	**1.09 (1.02–1.17)**	**1.22 (1.12–1.32)**	1 (Ref.)	1.00 (0.93–1.07)	1.05 (0.79–1.39)	1.03 (0.63–1.69)
	Mild	1.01 (0.97–1.05)	1 (Ref.)	1.04 (0.99–1.09)	1.07 (1.00–1.15)	0.97 (0.88–1.07)	1 (Ref.)	1.03 (0.77–1.37)	0.79 (0.45–1.40)
	Moderate	1.06 (0.98–1.14)	0.95 (0.90–1.01)	1 (Ref.)	1.03 (0.96–1.10)	0.81 (0.49–1.33)	1.04 (0.64–1.69)	1 (Ref.)	1.76 (0.93–3.34)
	Heavy	**1.08 (1.00–1.16)**	**0.89 (0.83–0.96)**	0.94 (0.89–1.00)	1 (Ref.)	0.64 (0.31–1.30)	0.85 (0.42–1.71)	0.95 (0.43–2.09)	1 (Ref.)
**Death**									
<65 years	None	1 (Ref.)	0.94 (0.89–1.00)	1.09 (1.00–1.20)	**1.26 (1.13–1.40)**	1 (Ref.)	1.01 (0.93–1.10)	0.97 (0.71–1.34)	1.01 (0.59–1.74)
	Mild	**1.22 (1.16–1.30)**	1 (Ref.)	**1.10 (1.03–1.17)**	**1.30 (1.19–1.42)**	1.03 (0.93–1.15)	1 (Ref.)	1.04 (0.82–1.31)	1.41 (0.95–2.09)
	Moderate	**1.38 (1.25–1.51)**	1.03 (0.97–1.10)	1 (Ref.)	1.06 (0.98–1.14)	**1.45 (1.01–2.06)**	0.95 (0.69–1.32)	1 (Ref.)	**1.71 (1.13–2.59)**
	Heavy	**1.50 (1.36–1.65)**	1.01 (0.93–1.10)	0.94 (0.88–1.00)	1 (Ref.)	0.81 (0.47–1.40)	0.63 (0.38–1.03)	1.00 (0.64–1.56)	1 (Ref.)
≥65	None	1 (Ref.)	**0.93 (0.89–0.97)**	1.04 (0.96–1.13)	**1.25 (1.15–1.36)**	1 (Ref.)	1.02 (0.95–1.09)	0.96 (0.71–1.30)	**1.62 (1.06–2.49)**

© 2022 Yoo JE et al. *JAMA Network Open.*

years	Mild	**1.28 (1.22–1.33)**	1 (Ref.)	**1.17 (1.10–1.24)**	**1.40 (1.30–1.52)**	**1.19 (1.06–1.34)**	1 (Ref.)	1.04 (0.74–1.48)	1.04 (0.58–1.84)
	Moderate	**1.40 (1.29–1.53)**	1.03 (0.96–1.11)	1 (Ref.)	**1.16 (1.06–1.26)**	1.22 (0.73–2.06)	1.16 (0.68–2.01)	1 (Ref.)	1.69 (0.78–3.68)
	Heavy	**1.38 (1.28–1.49)**	0.97 (0.90–1.05)	0.95 (0.89–1.02)	1 (Ref.)	1.25 (0.59–2.65)	0.96 (0.42–2.21)	1.18 (0.44–3.15)	1 (Ref.)

*Adjusted for sex, socioeconomic position (income level and place of residence), smoking status, comorbidities (hypertension, diabetes, dyslipidemia, chronic kidney disease, and chronic obstructive pulmonary disease), and Charlson comorbidity index

© 2022 Yoo JE et al. *JAMA Network Open.*

eTable 6. Adjusted Hazard Ratios* and 95% Confidence Intervals for Cancer According to Changes in Drinking Level by Sex

2009 \ 2011		None	Mild	Moderate	Heavy
**Alcohol-related cancers**					
Male	None	1 (Ref.)	**1.07 (1.03–1.12)**	**1.22 (1.13–1.32)**	**1.47 (1.35–1.60)**
	Mild	1.02 (0.97–1.06)	1 (Ref.)	**1.14 (1.08–1.19)**	**1.20 (1.12–1.29)**
	Moderate	**1.10 (1.02–1.19)**	0.99 (0.93–1.04)	1 (Ref.)	1.05 (0.99–1.12)
	Heavy	1.06 (0.97–1.15)	**0.92 (0.86–0.98)**	**0.90 (0.85–0.96)**	1 (Ref.)
Female	None	1 (Ref.)	1.01 (0.97–1.05)	0.96 (0.81–1.14)	0.83 (0.60–1.16)
	Mild	0.99 (0.94–1.04)	1 (Ref.)	1.03 (0.91–1.17)	0.84 (0.64–1.12)
	Moderate	0.86 (0.69–1.07)	0.84 (0.70–1.01)	1 (Ref.)	0.72 (0.52–1.00)
	Heavy	0.90 (0.62–1.31)	0.88 (0.63–1.22)	1.08 (0.78–1.51)	1 (Ref.)
**All cancers (except thyroid cancers)**					
Male	None	1 (Ref.)	0.99 (0.97–1.02)	**1.05 (1.00–1.10)**	**1.19 (1.13–1.25)**
	Mild	1.02 (0.99–1.05)	1 (Ref.)	**1.04 (1.01–1.07)**	**1.10 (1.05–1.15)**
	Moderate	**1.10 (1.05–1.15)**	0.98 (0.95–1.01)	1 (Ref.)	1.01 (0.97–1.05)
	Heavy	**1.08 (1.03–1.14)**	**0.92 (0.88–0.96)**	**0.95 (0.92–0.99)**	1 (Ref.)
Female	None	1 (Ref.)	1.00 (0.98–1.03)	0.97 (0.86–1.09)	1.01 (0.82–1.24)
	Mild	1.01 (0.97–1.05)	1 (Ref.)	1.03 (0.94–1.12)	0.96 (0.80–1.16)
	Moderate	0.99 (0.84–1.16)	0.99 (0.86–1.13)	1 (Ref.)	0.93 (0.74–1.17)
	Heavy	0.90 (0.69–1.18)	0.94 (0.74–1.20)	1.03 (0.80–1.32)	1 (Ref.)
**Death**					
Male	None	1 (Ref.)	**0.94 (0.91–0.97)**	**1.07 (1.01–1.14)**	**1.27 (1.19–1.35)**
	Mild	**1.26 (1.22–1.31)**	1 (Ref.)	**1.14 (1.09–1.19)**	**1.36 (1.28–1.44)**
	Moderate	**1.41 (1.32–1.50)**	1.03 (0.98–1.08)	1 (Ref.)	**1.11 (1.05–1.17)**
	Heavy	**1.43 (1.34–1.51)**	0.99 (0.93–1.04)	**0.94 (0.90–0.99)**	1 (Ref.)
Female	None	1 (Ref.)	**1.06 (1.00–1.12)**	1.03 (0.82–1.28)	**1.41 (1.01–1.98)**
	Mild	**1.09 (1.01–1.18)**	1 (Ref.)	1.05 (0.87–1.27)	1.27 (0.92–1.76)
	Moderate	1.30 (0.98–1.74)	0.96 (0.73–1.27)	1 (Ref.)	**1.72 (1.20–2.47)**
	Heavy	0.90 (0.59–1.37)	0.67 (0.44–1.02)	1.00 (0.67–1.50)	1 (Ref.)

*Adjusted for age, socioeconomic position (income level and place of residence), smoking status, comorbidities (hypertension, diabetes, dyslipidemia, chronic kidney disease, and chronic obstructive pulmonary disease), and Charlson comorbidity index

© 2022 Yoo JE et al. *JAMA Network Open.*

eTable 7. Adjusted Hazard Ratios and 95% Confidence Intervals for Cancer According to Changes in Drinking Level by Smoking Status in 2009 (Male)

2009 \ 2011		None	Mild	Moderate	Heavy
**Alcohol-related cancers**					
Never smoker	None	1 (Ref.)	1.04 (0.96–1.12)	**1.26 (1.09–1.47)**	**1.77 (1.51–2.08)**
	Mild	0.99 (0.92–1.07)	1 (Ref.)	1.08 (0.97–1.20)	**1.32 (1.14–1.54)**
	Moderate	1.09 (0.95–1.26)	0.95 (0.84–1.08)	1 (Ref.)	1.06 (0.91–1.24)
	Heavy	1.05 (0.92–1.19)	0.89 (0.77–1.02)	0.97 (0.85–1.10)	1 (Ref.)
Former smoker, <20 PY	None	1 (Ref.)	0.97 (0.87–1.07)	1.21 (1.00–1.46)	1.09 (0.84–1.43)
	Mild	**1.14 (1.03–1.26)**	1 (Ref.)	**1.34 (1.20–1.49)**	1.18 (0.96–1.44)
	Moderate	1.01 (0.80–1.27)	0.95 (0.83–1.08)	1 (Ref.)	1.00 (0.84–1.18)
	Heavy	1.02 (0.78–1.34)	0.92 (0.77–1.11)	0.85 (0.72–1.00)	1 (Ref.)
Former smoker, ≥20 PY	None	1 (Ref.)	1.04 (0.93–1.16)	1.09 (0.90–1.33)	**1.35 (1.10–1.64)**
	Mild	1.07 (0.95–1.20)	1 (Ref.)	1.13 (1.00–1.27)	1.14 (0.95–1.36)
	Moderate	**1.34 (1.11–1.62)**	1.02 (0.89–1.17)	1 (Ref.)	1.09 (0.94–1.27)
	Heavy	1.19 (0.99–1.44)	0.94 (0.80–1.11)	0.91 (0.79–1.04)	1 (Ref.)
Current smoker, <20 PY	None	1 (Ref.)	1.15 (1.02–1.30)	1.19 (0.97–1.47)	**1.63 (1.27–2.09)**
	Mild	0.93 (0.82–1.06)	1 (Ref.)	1.03 (0.92–1.17)	0.90 (0.72–1.12)
	Moderate	0.89 (0.70–1.15)	0.93 (0.82–1.05)	1 (Ref.)	0.86 (0.72–1.02)
	Heavy	1.29 (0.97–1.72)	1.09 (0.90–1.31)	1.03 (0.87–1.23)	1 (Ref.)
Current smoker, ≥20 PY	None	1 (Ref.)	**1.27 (1.14–1.41)**	**1.35 (1.16–1.58)**	**1.49 (1.27–1.75)**
	Mild	0.97 (0.87–1.08)	1 (Ref.)	**1.12 (1.02–1.24)**	**1.29 (1.14–1.47)**
	Moderate	1.07 (0.89–1.28)	1.04 (0.94–1.16)	1 (Ref.)	**1.15 (1.04–1.28)**
	Heavy	0.90 (0.74–1.09)	0.88 (0.78–1.00)	**0.86 (0.78–0.95)**	1 (Ref.)
**All cancers (except thyroid cancers)**					
Never smoker	None	1 (Ref.)	1.01 (0.96–1.05)	**1.14 (1.04–1.25)**	**1.41 (1.28–1.56)**
	Mild	1.02 (0.98–1.07)	1 (Ref.)	1.00 (0.94–1.07)	**1.20 (1.10–1.32)**
	Moderate	**1.10 (1.02–1.20)**	0.95 (0.88–1.02)	1 (Ref.)	0.99 (0.91–1.09)
	Heavy	**1.11 (1.02–1.20)**	**0.87 (0.80–0.95)**	0.93 (0.86–1.01)	1 (Ref.)
Former smoker, <20 PY	None	1 (Ref.)	1.01 (0.95–1.06)	1.07 (0.96–1.20)	1.03 (0.87–1.20)
	Mild	1.01 (0.95–1.07)	1 (Ref.)	1.12 (1.05–1.20)	1.04 (0.92–1.17)
	Moderate	1.11 (0.97–1.26)	**0.92 (0.85–0.99)**	1 (Ref.)	0.99 (0.89–1.09)
	Heavy	1.03 (0.87–1.22)	0.95 (0.85–1.07)	0.98 (0.89–1.08)	1 (Ref.)
Former smoker, ≥20 PY	None	1 (Ref.)	0.96 (0.91–1.02)	1.02 (0.91–1.14)	1.08 (0.96–1.22)
	Mild	1.06 (0.99–1.13)	1 (Ref.)	1.08 (1.01–1.15)	1.09 (0.98–1.20)
	Moderate	1.07 (0.95–1.19)	1.03 (0.95–1.11)	1 (Ref.)	1.00 (0.92–1.09)
	Heavy	1.04 (0.92–1.17)	0.92 (0.83–1.02)	0.93 (0.85–1.00)	1 (Ref.)
Current smoker, <20 PY	None	1 (Ref.)	0.99 (0.92–1.07)	0.93 (0.81–1.07)	1.15 (0.97–1.36)
	Mild	0.97 (0.90–1.05)	1 (Ref.)	0.99 (0.92–1.07)	0.95 (0.83–1.08)
	Moderate	0.93 (0.80–1.09)	0.95 (0.88–1.03)	1 (Ref.)	0.89 (0.80–1.00)
	Heavy	**1.24 (1.04–1.49)**	1.06 (0.94–1.20)	1.07 (0.96–1.19)	1 (Ref.)
Current smoker, ≥20 PY	None	1 (Ref.)	1.01 (0.95–1.07)	1.08 (0.99–1.17)	**1.18 (1.08–1.30)**
	Mild	1.00 (0.94–1.07)	1 (Ref.)	1.01 (0.96–1.07)	**1.09 (1.00–1.17)**
	Moderate	**1.16 (1.05–1.28)**	1.02 (0.96–1.08)	1 (Ref.)	**1.07 (1.00–1.14)**
	Heavy	1.01 (0.90–1.13)	0.92 (0.86–1.00)	0.95 (0.90–1.01)	1 (Ref.)
**Death**					
Never	None	1 (Ref.)	**0.92 (0.87–0.98)**	1.03 (0.91–1.16)	**1.37 (1.21–1.55)**

© 2022 Yoo JE et al. *JAMA Network Open.*

smoker	Mild	**1.30 (1.23–1.37)**	1 (Ref.)	**1.19 (1.09–1.30)**	**1.61 (1.43–1.81)**
	Moderate	**1.46 (1.30–1.64)**	1.09 (0.98–1.22)	1 (Ref.)	1.07 (0.93–1.23)
	Heavy	**1.39 (1.26–1.54)**	0.97 (0.87–1.09)	1.00 (0.89–1.11)	1 (Ref.)
Former smoker, <20 PY	None	1 (Ref.)	**0.88 (0.81–0.95)**	1.04 (0.87–1.23)	**1.34 (1.09–1.64)**
	Mild	**1.26 (1.15–1.38)**	1 (Ref.)	1.14 (1.02–1.28)	**1.23 (1.02–1.48)**
	Moderate	**1.39 (1.14–1.69)**	1.08 (0.94–1.23)	1 (Ref.)	1.11 (0.94–1.32)
	Heavy	1.24 (0.99–1.54)	0.88 (0.73–1.05)	**0.84 (0.71–0.99)**	1 (Ref.)
Former smoker, ≥20 PY	None	1 (Ref.)	**0.92 (0.85–0.99)**	1.01 (0.87–1.16)	1.13 (0.98–1.32)
	Mild	**1.29 (1.19–1.41)**	1 (Ref.)	1.10 (0.99–1.22)	**1.29 (1.12–1.48)**
	Moderate	**1.43 (1.23–1.67)**	1.10 (0.98–1.24)	1 (Ref.)	**1.23 (1.08–1.41)**
	Heavy	**1.46 (1.27–1.67)**	0.95 (0.83–1.09)	0.91 (0.81–1.03)	1 (Ref.)
Current smoker, <20 PY	None	1 (Ref.)	1.02 (0.93–1.12)	1.07 (0.91–1.27)	1.18 (0.96–1.45)
	Mild	**1.13 (1.02–1.25)**	1 (Ref.)	1.07 (0.97–1.19)	1.15 (0.97–1.36)
	Moderate	1.06 (0.87–1.28)	0.91 (0.81–1.01)	1 (Ref.)	1.02 (0.89–1.18)
	Heavy	**1.44 (1.17–1.76)**	1.09 (0.94–1.27)	1.09 (0.95–1.26)	1 (Ref.)
Current smoker, ≥20 PY	None	1 (Ref.)	1.01 (0.94–1.08)	**1.17 (1.05–1.31)**	**1.30 (1.16–1.45)**
	Mild	**1.15 (1.06–1.24)**	1 (Ref.)	**1.12 (1.03–1.21)**	**1.34 (1.21–1.48)**
	Moderate	**1.37 (1.21–1.56)**	0.99 (0.91–1.08)	1 (Ref.)	**1.12 (1.03–1.22)**
	Heavy	**1.34 (1.19–1.51)**	0.99 (0.90–1.09)	**0.90 (0.83–0.97)**	1 (Ref.)

PY, pack-years

*Adjusted for age, socioeconomic position (income level and place of residence), smoking status, comorbidities (hypertension, diabetes, dyslipidemia, chronic kidney disease, and chronic obstructive pulmonary disease), and Charlson comorbidity index

© 2022 Yoo JE et al. *JAMA Network Open.*

eTable 8. Adjusted Hazard Ratios and 95% Confidence Intervals for Cancer According to Changes in Drinking Level by Smoking Status in 2009 (Female)

2009 \ 2011		None	Mild	Moderate	Heavy
**Alcohol-related cancers**					
Never smoker	None	1 (Ref.)	1.01 (0.96–1.05)	0.94 (0.78–1.14)	0.92 (0.64–1.33)
	Mild	1.00 (0.95–1.06)	1 (Ref.)	1.05 (0.92–1.20)	0.89 (0.65–1.22)
	Moderate	0.90 (0.71–1.14)	0.87 (0.70–1.07)	1 (Ref.)	0.78 (0.52–1.16)
	Heavy	1.02 (0.65–1.59)	0.99 (0.65–1.52)	1.09 (0.70–1.71)	1 (Ref.)
Former smoker, <20 PY	None	1 (Ref.)	1.16 (0.82–1.64)	1.08 (0.44–2.64)	N/A
	Mild	**0.57 (0.35–0.93)**	1 (Ref.)	1.07 (0.62–1.86)	0.81 (0.26–2.56)
	Moderate	1.55 (0.48–5.02)	1.25 (0.51–3.10)	1 (Ref.)	0.32 (0.04–2.65)
	Heavy	1.32 (0.32–5.48)	1.00 (0.32–3.16)	1.42 (0.50–4.02)	1 (Ref.)
Former smoker, ≥20 PY	None	1 (Ref.)	1.94 (0.89–4.23)	0.80 (0.11–5.91)	N/A
	Mild	0.71 (0.16–3.13)	1 (Ref.)	0.99 (0.07–13.40)	N/A
	Moderate	N/A	N/A	1 (Ref.)	N/A
	Heavy	N/A	N/A	N/A	1 (Ref.)
Current smoker, <20 PY	None	1 (Ref.)	0.90 (0.68–1.20)	1.26 (0.77–2.06)	1.03 (0.46–2.33)
	Mild	0.90 (0.64–1.26)	1 (Ref.)	0.92 (0.60–1.39)	0.89 (0.41–1.91)
	Moderate	0.52 (0.23–1.16)	**0.60 (0.38–0.95)**	1 (Ref.)	0.63 (0.32–1.22)
	Heavy	0.41 (0.09–1.79)	0.71 (0.32–1.56)	1.15 (0.60–2.21)	1 (Ref.)
Current smoker, ≥20 PY	None	1 (Ref.)	0.85 (0.43–1.69)	0.28 (0.04–2.03)	N/A
	Mild	1.25 (0.51–3.07)	1 (Ref.)	0.53 (0.12–2.44)	N/A
	Moderate	0.93 (0.09–9.37)	1.90 (0.45–8.04)	1 (Ref.)	1.28 (0.25–6.58)
	Heavy	0.56 (0.07–4.87)	0.32 (0.04–2.55)	0.67 (0.20–2.20)	1 (Ref.)
**All cancers (except thyroid cancers)**					
Never smoker	None	1 (Ref.)	1.01 (0.98–1.04)	0.96 (0.84–1.10)	1.13 (0.90–1.42)
	Mild	1.02 (0.98–1.06)	1 (Ref.)	1.03 (0.93–1.13)	1.04 (0.84–1.27)
	Moderate	0.99 (0.83–1.18)	1.00 (0.85–1.16)	1 (Ref.)	0.94 (0.71–1.24)
	Heavy	0.99 (0.72–1.37)	1.11 (0.82–1.50)	1.10 (0.79–1.53)	1 (Ref.)
Former smoker, <20 PY	None	1 (Ref.)	0.93 (0.72–1.21)	0.93 (0.48–1.81)	0.26 (0.04–1.84)
	Mild	**0.67 (0.47–0.96)**	1 (Ref.)	1.16 (0.77–1.75)	0.79 (0.32–1.92)
	Moderate	1.45 (0.62–3.42)	1.08 (0.56–2.07)	1 (Ref.)	0.68 (0.22–2.07)
	Heavy	2.13 (0.74–6.15)	0.92 (0.34–2.49)	1.25 (0.50–3.13)	1 (Ref.)
Former smoker, ≥20 PY	None	1 (Ref.)	1.59 (0.90–2.82)	0.41 (0.06–2.97)	0.63 (0.09–4.62)
	Mild	1.12 (0.39–3.27)	1 (Ref.)	1.63 (0.30–8.93)	N/A
	Moderate	N/A	N/A	1 (Ref.)	N/A
	Heavy	N/A	N/A	N/A	1 (Ref.)
Current smoker, <20 PY	None	1 (Ref.)	**0.82 (0.68–0.99)**	1.18 (0.84–1.66)	0.72 (0.37–1.39)
	Mild	0.83 (0.66–1.05)	1 (Ref.)	1.02 (0.78–1.35)	0.92 (0.55–1.55)
	Moderate	0.81 (0.47–1.40)	0.80 (0.57–1.13)	1 (Ref.)	0.82 (0.50–1.33)
	Heavy	0.58 (0.24–1.41)	0.63 (0.35–1.14)	0.93 (0.56–1.55)	1 (Ref.)
Current smoker, ≥20 PY	None	1 (Ref.)	0.84 (0.54–1.29)	0.60 (0.25–1.46)	0.90 (0.37–2.21)
	Mild	1.35 (0.78–2.32)	1 (Ref.)	0.92 (0.39–2.15)	0.42 (0.10–1.76)
	Moderate	1.92 (0.50–7.39)	2.84 (0.99–8.14)	1 (Ref.)	2.31 (0.76–7.03)
	Heavy	0.20 (0.03–1.54)	0.44 (0.13–1.53)	0.71 (0.30–1.67)	1 (Ref.)
**Death**					
Never	None	1 (Ref.)	1.06 (1.00–1.13)	1.03 (0.80–1.33)	**1.53 (1.03–2.26)**

© 2022 Yoo JE et al. *JAMA Network Open.*

smoker	Mild	1.09 (1.00–1.18)	1 (Ref.)	1.00 (0.80–1.26)	**1.49 (1.04–2.15)**
	Moderate	1.11 (0.80–1.54)	0.83 (0.60–1.15)	1 (Ref.)	1.11 (0.66–1.85)
	Heavy	0.93 (0.55–1.57)	0.86 (0.51–1.46)	1.11 (0.63–1.95)	1 (Ref.)
Former smoker, <20 PY	None	1 (Ref.)	1.01 (0.68–1.49)	0.44 (0.06–3.15)	0.89 (0.13–6.39)
	Mild	1.30 (0.76–2.21)	1 (Ref.)	1.25 (0.52–2.98)	N/A
	Moderate	1.64 (0.39–6.86)	1.19 (0.37–3.85)	1 (Ref.)	1.07 (0.19–5.96)
	Heavy	2.26 (0.33–15.61)	0.22 (0.02–2.35)	0.19 (0.01–2.75)	1 (Ref.)
Former smoker, ≥20 PY	None	1 (Ref.)	1.37 (0.72–2.61)	0.68 (0.09–4.96)	1.04 (0.14–7.57)
	Mild	2.78 (0.55–13.99)	1 (Ref.)	**12.53 (1.25–125.92)**	N/A
	Moderate	N/A	N/A	1 (Ref.)	N/A
	Heavy	N/A	N/A	N/A	1 (Ref.)
Current smoker, <20 PY	None	1 (Ref.)	0.87 (0.68–1.12)	1.20 (0.71–2.05)	1.08 (0.40–2.90)
	Mild	1.05 (0.75–1.46)	1 (Ref.)	1.12 (0.69–1.80)	0.60 (0.19–1.90)
	Moderate	**2.40 (1.14–5.06)**	1.27 (0.67–2.43)	1 (Ref.)	**3.86 (1.98–7.54)**
	Heavy	0.78 (0.25–2.43)	0.30 (0.10–0.92)	1.13 (0.56–2.27)	1 (Ref.)
Current smoker, ≥20 PY	None	1 (Ref.)	1.17 (0.79–1.74)	0.60 (0.19–1.88)	1.08 (0.34–3.40)
	Mild	0.91 (0.51–1.62)	1 (Ref.)	0.81 (0.28–2.37)	1.34 (0.50–3.59)
	Moderate	0.80 (0.08–8.33)	1.70 (0.35–8.20)	1 (Ref.)	1.78 (0.32–9.87)
	Heavy	1.19 (0.11–13.13)	0.82 (0.13–5.04)	0.47 (0.09–2.55)	1 (Ref.)

PY, pack-years

*Adjusted for age, socioeconomic position (income level and place of residence), smoking status, comorbidities (hypertension, diabetes, dyslipidemia, chronic kidney disease, and chronic obstructive pulmonary disease), and Charlson comorbidity index

© 2022 Yoo JE et al. *JAMA Network Open.*

eTable 9. Associations Between Changes in Drinking Level and Cancer (Sustained Non-Drinker as a Referent)

Alcohol consumption status		aHR (95% CI)*
2009	2011	
**Alcohol-related cancers**		
None	None	1 (Ref.)
	Mild	**1.11 (1.07–1.14)**
	Moderate	**1.19 (1.11–1.28)**
	Heavy	**1.44 (1.32–1.56)**
Mild	None	**1.11 (1.08–1.14)**
	Mild	**1.07 (1.04–1.09)**
	Moderate	**1.15 (1.10–1.20)**
	Heavy	**1.24 (1.16–1.33)**
Moderate	None	**1.35 (1.27–1.44)**
	Mild	**1.16 (1.11–1.20)**
	Moderate	**1.13 (1.09–1.18)**
	Heavy	**1.22 (1.16–1.29)**
Heavy	None	**1.68 (1.56–1.80)**
	Mild	**1.34 (1.26–1.42)**
	Moderate	**1.26 (1.20–1.33)**
	Heavy	**1.41 (1.36–1.47)**
**All cancers (except thyroid cancers)**		
None	None	1 (Ref.)
	Mild	**0.96 (0.93–0.99)**
	Moderate	**1.10 (1.05–1.15)**
	Heavy	**1.17 (1.09–1.25)**
Mild	None	1.05 (0.98–1.14)
	Mild	0.98 (0.93–1.03)
	Moderate	1.05 (0.98–1.12)
	Heavy	1.05 (0.98–1.14)
Moderate	None	0.98 (0.93–1.04)
	Mild	1.04 (0.98–1.11)
	Moderate	1.03 (0.95–1.12)
	Heavy	0.91 (0.85–0.97)
Heavy	None	**0.91 (0.86–0.96)**
	Mild	1.04 (0.96–1.12)
	Moderate	**0.92 (0.86–0.98)**
	Heavy	**0.91 (0.86–0.97)**

aHR, adjusted hazard ratio; CI, confidence interval

*adjusted for age, sex, socioeconomic position (income level and place of residence), smoking status, physical activity, comorbidities (hypertension, diabetes, dyslipidemia, chronic kidney disease, and chronic obstructive pulmonary disease), and Charlson comorbidity index

© 2022 Yoo JE et al. *JAMA Network Open.*

eFigure 1. Flow Chart of the Study Population

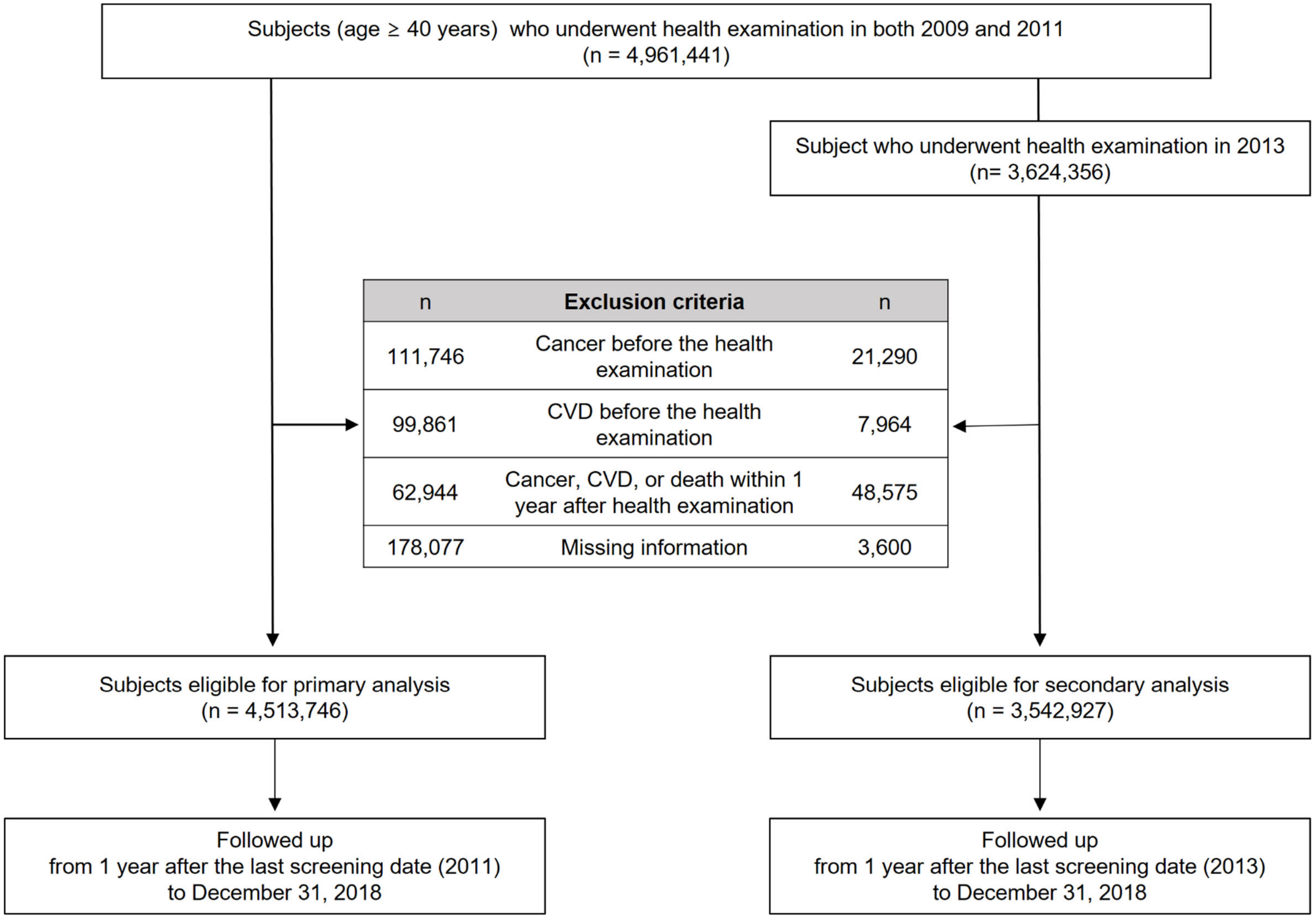

© 2022 Yoo JE et al. *JAMA Network Open.*

eFigure 2. The Risk of Site-Specific Cancer According to Changes in Drinking Level Between 2009 and 2011

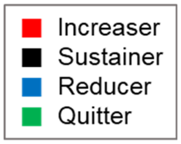

A. Lip, oral cavity and pharynx

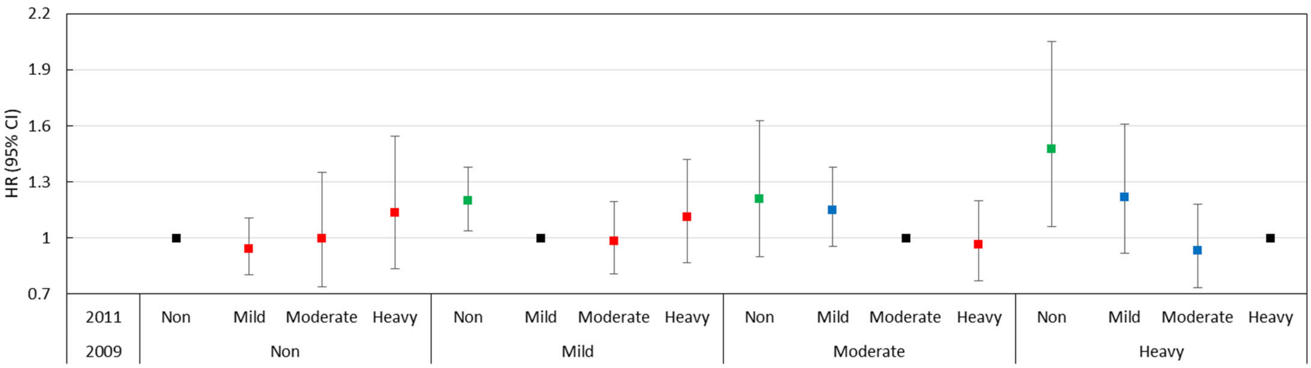

B. Esophagus

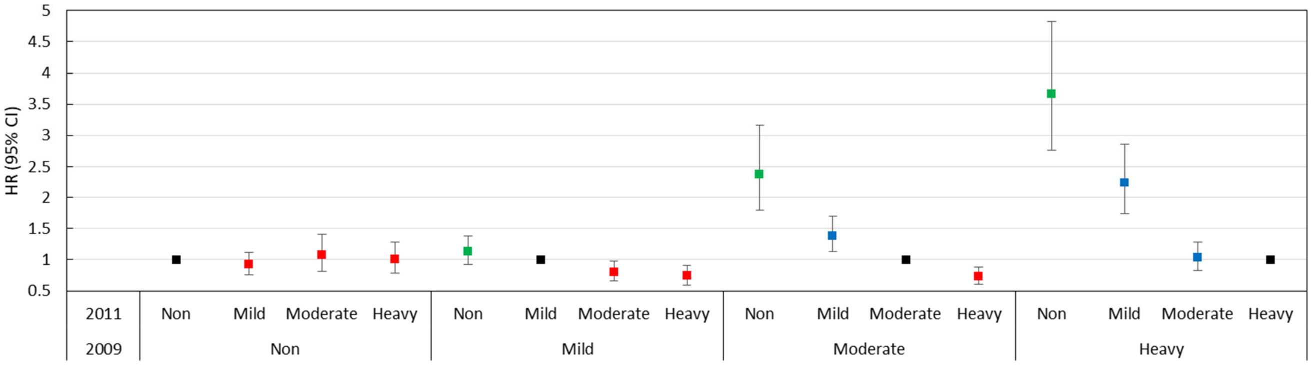

C. Stomach

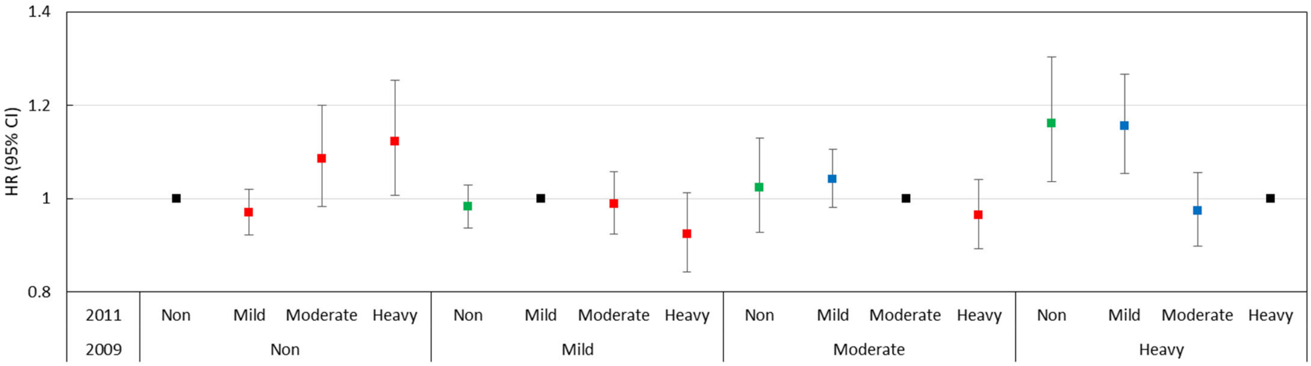

© 2022 Yoo JE et al. *JAMA Network Open.*

D. Colorectum

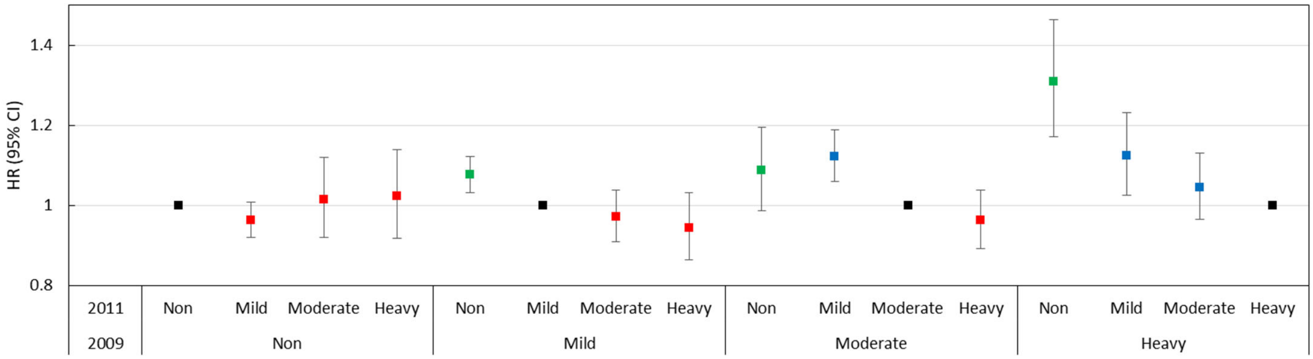

E. Liver

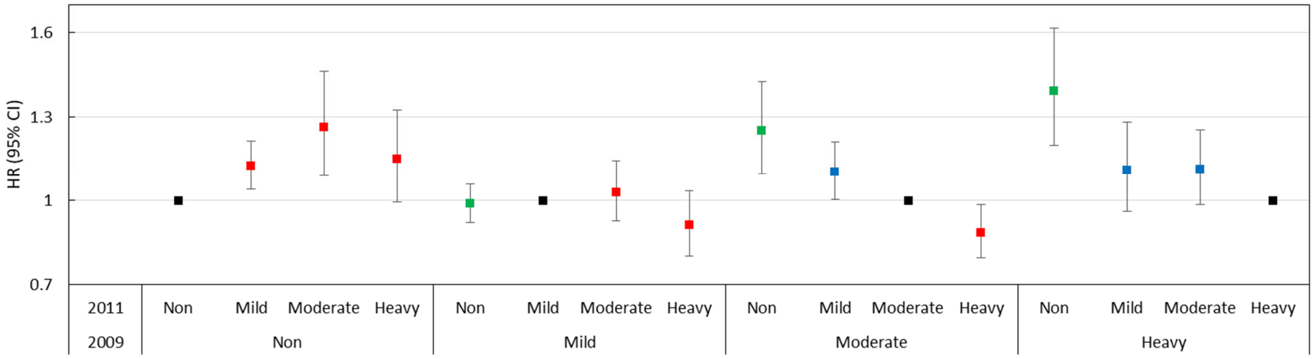

F. Gallbladder

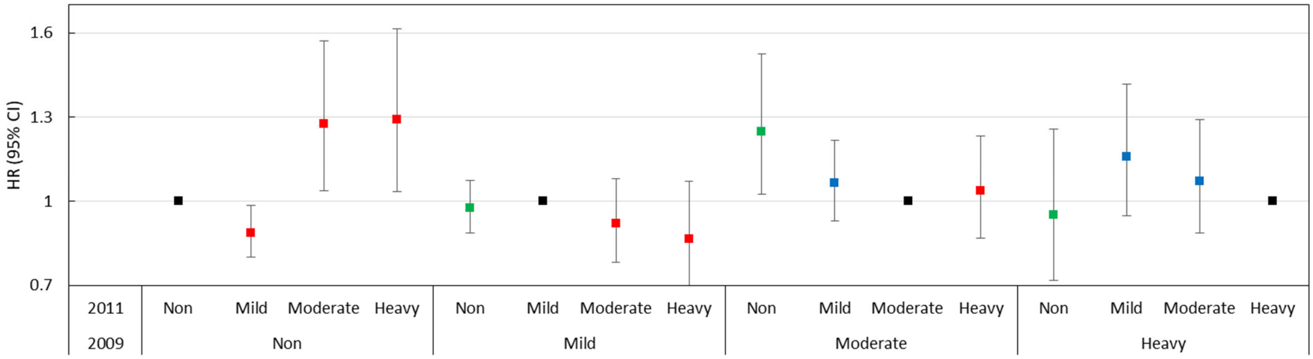

G. Pancreas

© 2022 Yoo JE et al. *JAMA Network Open.*

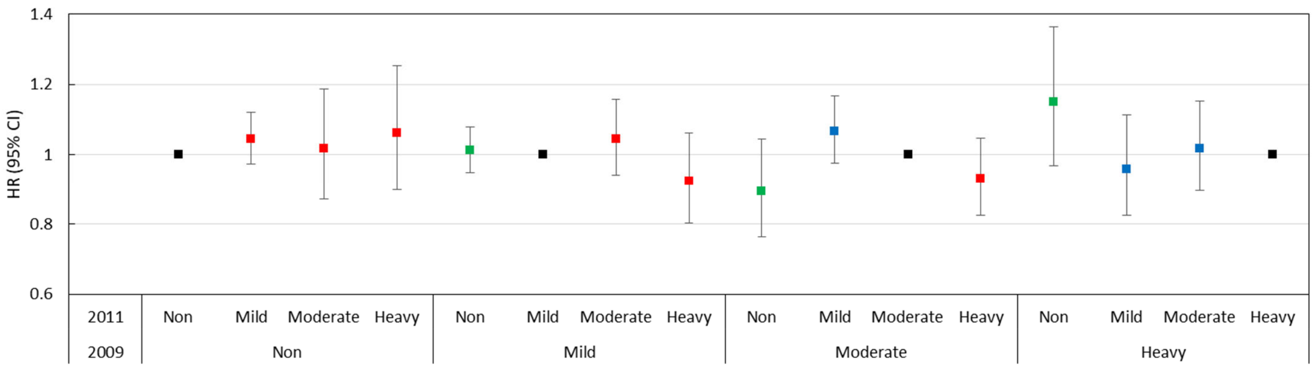

H. Larynx

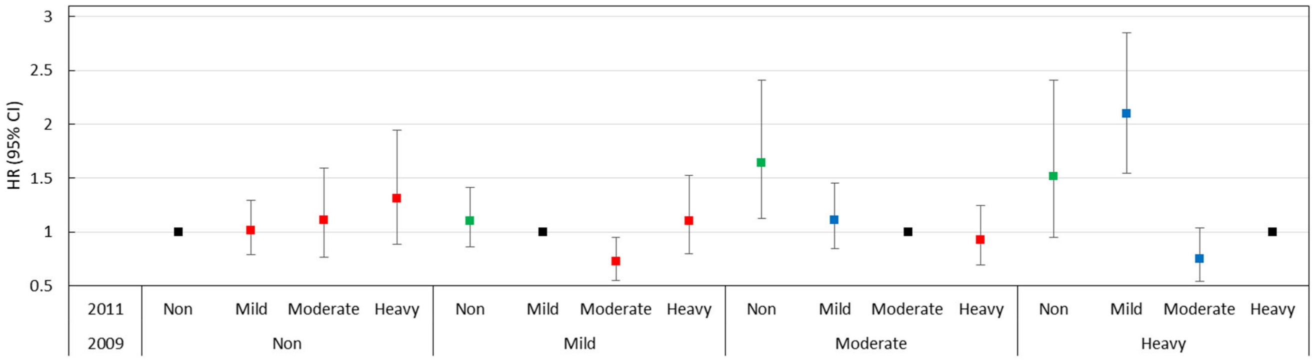

I. Lung

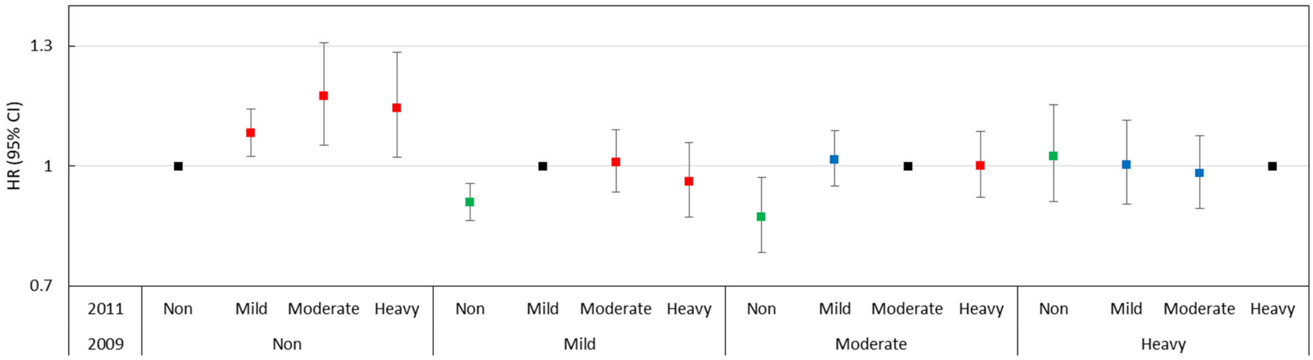

J. Breast

© 2022 Yoo JE et al. *JAMA Network Open.*

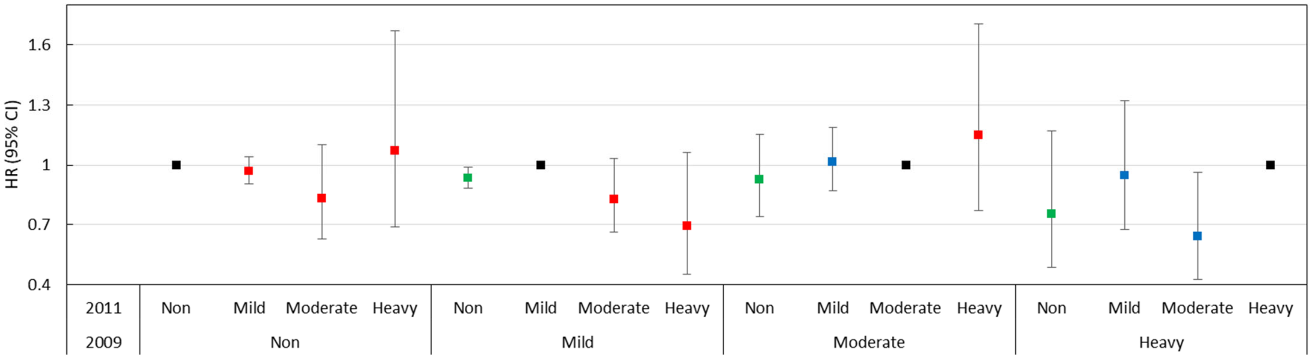

K. Cervix uteri

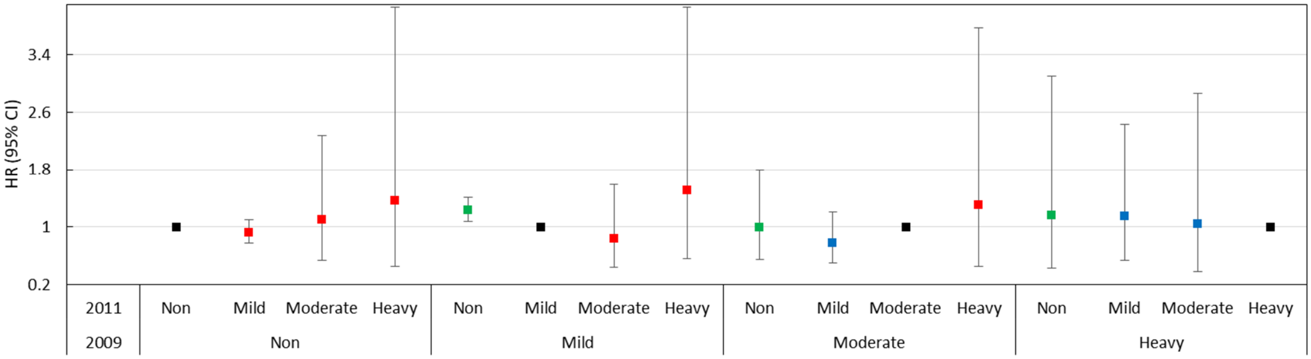

L. Corpus uteri

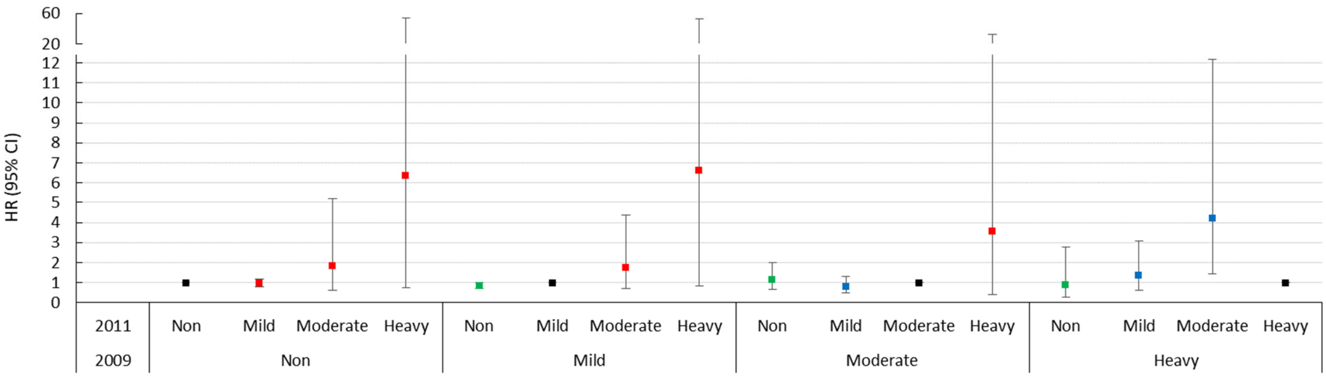

M. Ovary

© 2022 Yoo JE et al. *JAMA Network Open.*

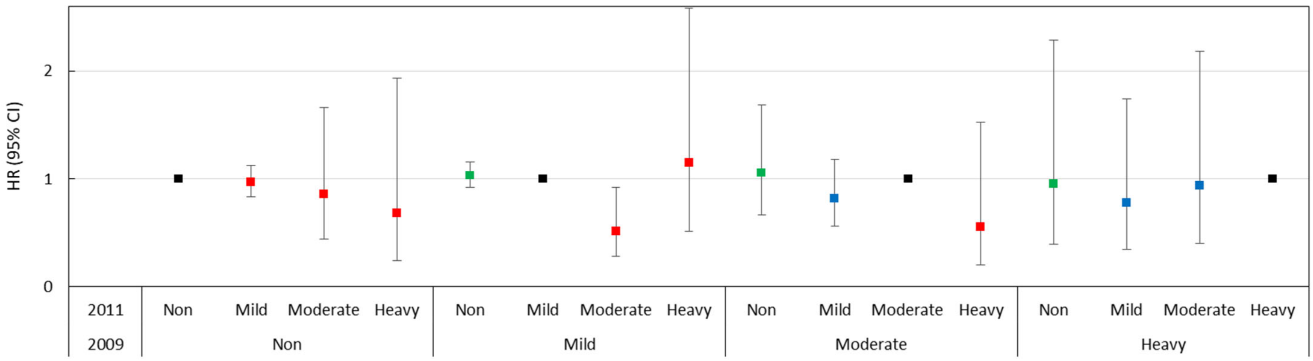

N. Prostate

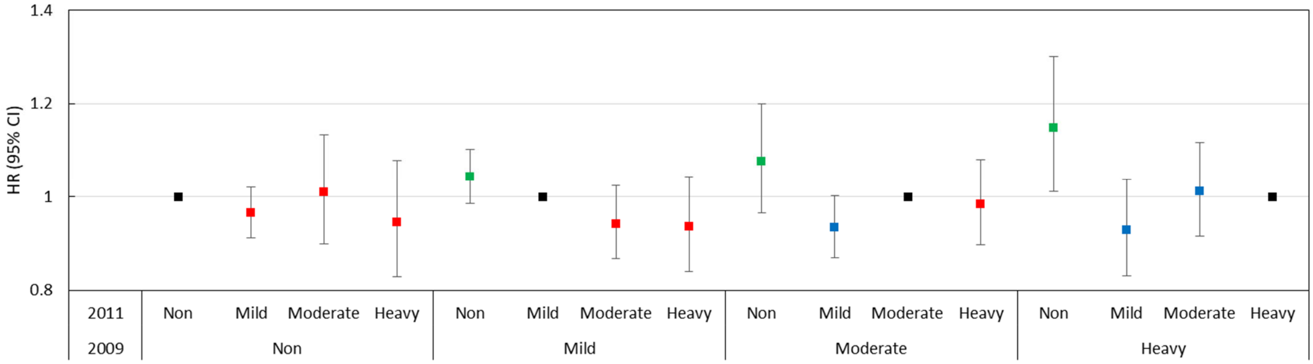

O. Kidney

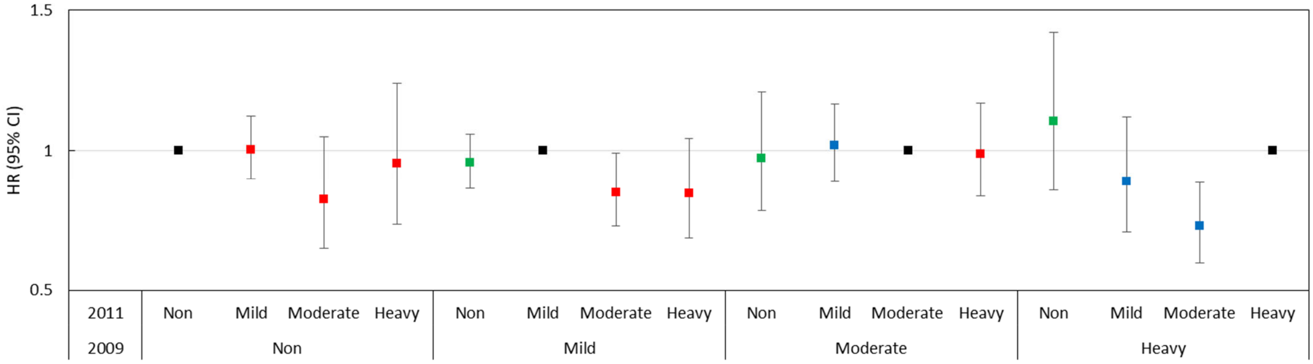

P. Bladder

© 2022 Yoo JE et al. *JAMA Network Open.*

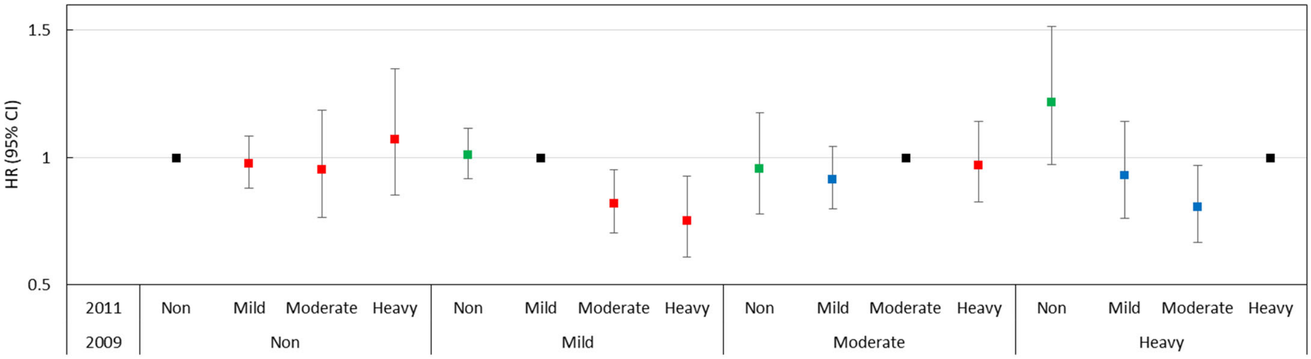

Q. Brain and CNS

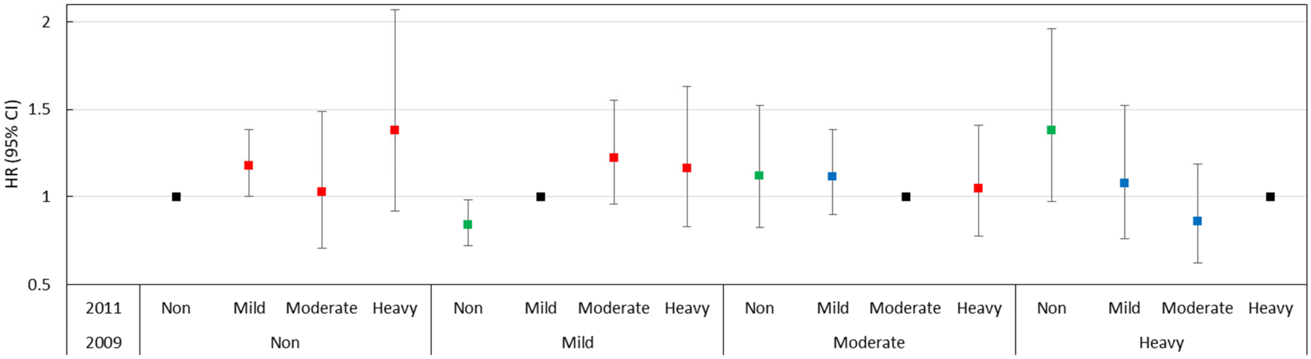

R. Hodgkin lymphoma

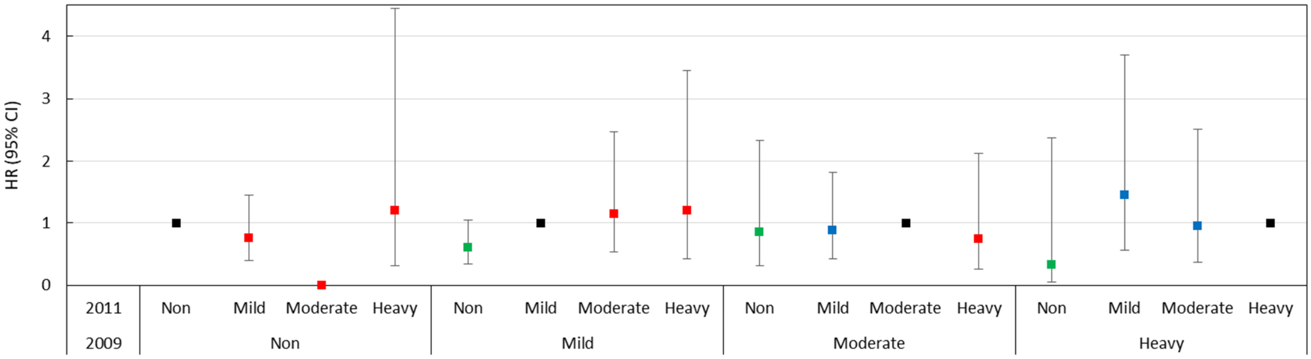

S. Non-Hodgkin lymphoma

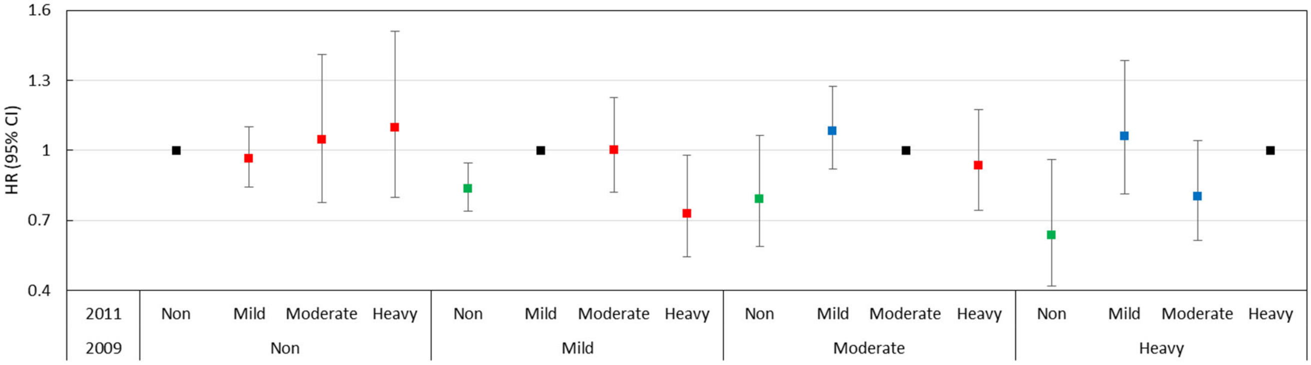

© 2022 Yoo JE et al. *JAMA Network Open.*

T. Multiple myeloma

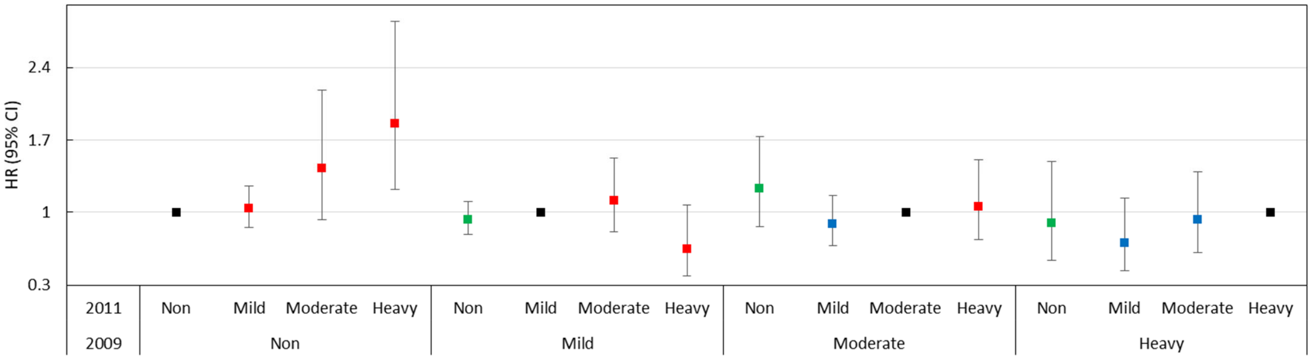

U. Leukemia

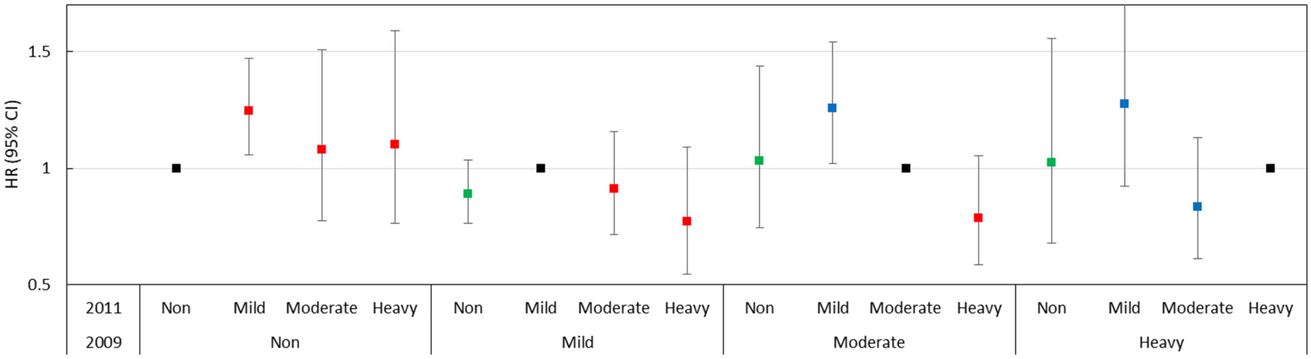

HRs are adjusted for age, sex, socioeconomic position (income level and place of residence), smoking status, physical activity, comorbidities (hypertension, diabetes, dyslipidemia, chronic kidney disease, and chronic obstructive pulmonary disease), and Charlson comorbidity index

© 2022 Yoo JE et al. *JAMA Network Open.*
